# Genome-Wide Identification and Analysis of Lipases in Fig Wasps (Chalcidoidea, Hymenoptera)

**DOI:** 10.3390/insects13050407

**Published:** 2022-04-24

**Authors:** Xianqin Wei, Jiaxing Li, Tao Wang, Jinhua Xiao, Dawei Huang

**Affiliations:** College of Life Sciences, Nankai University, Tianjin 300071, China; weixq@nankai.edu.cn (X.W.); 1120200483@mail.nankai.edu.cn (J.L.); w970528354@163.com (T.W.)

**Keywords:** neutral lipase, acid lipase, tandem duplication, phylogeny, catalytic triad

## Abstract

**Simple Summary:**

Lipases are a large family of enzymes involved in lipid metabolism. Lipids play diverse roles in insect growth and responses to environmental stimuli. Fig wasps are a polyphyletic assemblage of Chalcidoidea that develop in the inflorescences of fig trees. Based on whether they can pollinate, they are separated into pollinator fig wasp (PFW) and non-pollinating fig wasp (NPFW). In this study, we conducted a genome-wide screening of lipases in the 12 fig wasp genomes using bioinformatics tools, including seven PFWs and five NPFWs. In total, 481 lipase genes were identified with the neutral and acid lipases as the most numerous families. NPFWs had significantly more lipases than PFWs. Tandem duplication accounted for the expansion of the gene family. Phylogenetic analysis indicated that the lipase genes were conserved. This study provided evidence of insect metabolism to understand the obligate mutualism between figs and fig wasps. Our results will facilitate the understanding of the molecular mechanism of how lipase proteins contribute to the distinctions of life histories between PFWs and NPFWs.

**Abstract:**

Lipases are the main enzymes involved in lipid metabolism. However, the characteristics of lipases in insects were scarcely investigated. Here, we screened the recently sequenced genomes of 12 fig wasp species consisting of seven pollinator fig wasps (PFWs) and five non-pollinating fig wasps (NPFWs) for the six major lipase gene families. In total, 481 lipase genes were identified, and the two most numerous families were the neutral and acid lipases. Tandem duplication accounted for the expansion of the gene family. NPFWs had significantly more lipases than PFWs. A significant gene family contraction occurred in the clade of PFWs. The difference of lipases between NPFWs and PFWs might contribute to their distinction in life histories and feeding regimes. Phylogenetic analysis showed that the lipase genes of each fig wasp species was almost equally distributed in each clade, indicating that the lipase genes were conserved. The gene structures were similar within each clade, while they were different among clades. Most of the neutral and acid lipases were signal peptides and located extracellularly. The pathways of lipases involved were predicted. This genome-wide study provides a systematic analysis of lipase gene families in 12 hymenopteran insects and further insights towards understanding the potential functions of lipases.

## 1. Introduction

Lipids and their metabolites play diverse roles in insect growth as well as responses to environmental stimuli [1,2,3]. Lipases are a large family of enzymes involved in lipid metabolism [4]. Lipases are generally defined as triacylglycerol (TAG) hydrolases (EC 3.1.1.3), which play key roles in insect lipid acquisition, storage and mobilization [5]. Most lipases from all organisms can be divided into six families, including the neutral (PF00151), acid (PF04083), lipase2 (PF01674), lipase3 (PF01764), lipase with motif Gly-Asp-Ser-(Leu) (GDSL, PF00657) and hormone sensitive lipase (HSL, PF06350) [6,7]. The neutral lipases are able to hydrolyze neutral lipids such as TAGs, diacylglycerides (DAGs), monoacylglycerols (MAGs), the sn-1 position in phospholipids and galactolipids [8]. The acid lipases predominantly hydrolyze TAGs and cholesterol esters [9]. The acid lipases functioned at an acidic pH, whereas the activity of human pancreatic lipase from the neutral family has a pH optimum of 8 [9]. The GDSL family can hydrolyze fatty acids from TAGs, DAGs and MAGs, as well as from the sn-2 position of phospholipids, and can also hydrolyze carboxyl ester or thiol ester substrates [10]. The lipase2 and lipase3 family members can hydrolyze TAGs or carboxyl esters with varying fatty acid constituents [7]. The hormone-sensitive lipases hydrolyze TAGs and cholesterol esters but are under significant hormonal and neuronal control through reversible cAMP-dependent phosphorylation of a serine residue [11,12].

The lipase gene families were characterized in several insects. Horne, et al. [13] investigated the lipase of five insect species and found that the total number of the lipases varied two-fold. Five lipase gene families were identified in insects, among which the neutral and acid lipases were the two most abundant families, occupying from 69.23% (*Apis mellifera*) to 92.86% (*Drosophila melanogaster*) of the total number of lipases [13,14]. Neutral lipase family genes in *Drosophila* underwent multiple tandem duplication events leading to the expansion of gene family size [15]. However, most of the insect lipases lack TAG hydrolysis activity, even though the acid lipases had intact cap domains required for TAG hydrolysis [14]. Wang, et al. [14] also found the loss of catalytic activities and weak TAG hydrolytic activities of lipases in the venom of the parasitoid wasp *Pteromalus puparum*. Thus, the biological significance of lipase diversity remains unclear.

Fig wasps are a polyphyletic assemblage of Chalcidoidea that develop in the inflorescences of fig trees [16]. Based on whether they can pollinate, they are separated into pollinating fig wasp (PFW) and non-pollinating fig wasp (NPFW) [17]. The PFWs complete their life cycles mainly within the syconia by utilizing the nutrition of the female floret ovaries inside the syconia [18]. However, most of the NPFWs oviposit from the outside using the ovipositor to penetrate the syconium [19,20]. According to their special feeding habitats, the NPFWs can be categorized into gall-makers, parasitoids and inquilines [21]. Parasitoid larvae feed on the larvae of pollinators or gall-makers, and inquilines feed on the gall tissue induced by the larvae of either gall-makers or pollinators [22]. The diversified feeding regimes of fig wasps may be related to the lipases, which has not been studied yet. Additionally, the feeding regimes affected longevity in most species, as reported in *Ficus racemosa* [23]. The NPFWs had a longer lifespan than the PFWs, thus the NPFWs need more energy to support their long-life histories. We suspected that the NPFWs had more lipases than PFWs.

In this study, we manually annotated all the lipase in the genomes of 12 fig wasp species, analyzed the gene structures and compared the amino acid sequences encoded by these genes. We investigated the gene duplication events and conducted the gene expansions and contractions analysis. The functional analysis was also attempted, and the tertiary structures were predicted. This study was the first comprehensive analysis of the lipase gene families in fig wasps. Our results provide useful information for further probing the molecular mechanism of how lipase proteins contribute to the distinctions of life histories between PFWs and NPFWs.

## 2. Materials and Methods

### 2.1. Manual Annotation of the Lipase Gene Family

We used the seven lipase genes from *Drosophila melanogaster* as query sequences to make local tblastn in the genomes of 12 fig wasp species, including seven pollinators (*Eupristina koningsbergeri*, *Platyscapa corneri*, *Kradibia gibbosae*, *Ceratosolen fusciceps*, *Ceratosolen solmsi*, *Dolichoris vasculosae*, and *Wiebesia pumilae*), and five NPFWs (*Apocrypta bakeri*, *Philotrypesis tridentata*, *Sycobia* sp.2, *Sycophila* sp.2, and *Sycophaga agraensis*) (Table S1). Except for the data of *C. solmsi* downloaded from National Center for Biotechnology Information (NCBI, https://www.ncbi.nlm.nih.gov, accessed on 26 August 2021) under the project accession number PRJNA277475, the genomes and transcriptomes of other 11 fig wasps were deposited to NCBI under the project accession numbers PRJNA641212 and PRJNA494992 [24]. The gene sequences and structures were verified by an Integrative Genomics Viewer (IGV) (https://igv.org, accessed on 26 August 2021) [25]. Softberry (http://www.softberry.com, accessed on 26 August 2021) [26] was used to predict the gene structures of the lipase genes which lacked certain information from the IGV. All predicted lipase genes were manually validated by blastp (https://blast.ncbi.nlm.nih.gov/Blast.cgi?PROGRAM=blastp&PAGE_TYPE=BlastSearch&LINK_LOC=blasthome, accessed on 26 August 2021). Finally, we obtained the amino acid sequences and nucleic acid sequences of lipase for the 12 fig wasps. Additionally, the neutral and acid lipase genes in *Acyrthosiphon pisum* and *Daphnia pulex* were manually annotated with the same approach and used for the following analysis of gene family expansion and contraction.

### 2.2. Phylogenetic Analysis

Phylogenetic trees were constructed for neutral and acid lipase protein sequences of 12 fig wasps. We performed phylogenetic analysis in PhyloSuite (https://dongzhang0725.github.io, accessed on 6 September 2021) [27]. Firstly, we made multiple sequence alignments with MAFFT software (https://mafft.cbrc.jp/alignment/software/, accessed on 6 September 2021) [28]. According to the Akaike Information Criterion (AIC), ModelFinder (http://www.iqtree.org/doc/Tutorial#choosing-the-right-substitution-model, accessed on 6 September 2021) [29] revealed WAG + F + R10 and JTT + F + R7 as the best models of molecular evolution with the best fit to the neutral and acid lipase data, respectively, and Maximum-likelihood (ML) trees were constructed with the IQ-TREE (http://www.iqtree.org, accessed on 6 September 2021) [30,31]. Phylogenetic trees were visualized with the online tool iTOL v6 [32].

### 2.3. Gene Family Expansions and Contractions

In order to understand gene family expansions and contractions, we used the analytical approach implemented in the Computational Analysis of gene Family Evolution (CAFÉ) 4.2.1 [33]. CAFÉ employs a random birth-death process to model changes in gene family size while accounting for phylogenetic relationships. Based on lambda values (the probability of gene gain and loss per unit of time during species evolution) with default parameters, the divergence tree constructed by Xiao, et al. [24] was applied in this analysis.

### 2.4. Gene Structure Analysis

The exon/intron composition of a gene was estimated using GSD 2.0 (http://gsds.gao-lab.org/index.php, accessed on 14 September 2021) [34]. To search the tandem duplication events, collinearity analysis was conducted using MCScanX (https://github.com/wyp1125/MCScanx, accessed on 14 September 2021) [35], and the location of the lipase gene on the scaffold was determined by TBtools (https://github.com/CJ-Chen/TBtools, accessed on 14 September 2021) [36]. The motif structures were determined by MEME Suite 5.3.2 (http://meme-suite.org/tools/meme, accessed on 14 September 2021) [37].

### 2.5. Identification of Catalytic Triads in Lipases

The neutral and acid lipases require a catalytic triad consisting of Ser-Asp-His residues to drive the catalytic mechanisms [7]. Sequences of well-recognized canine pancreatic (NP_001003319) and gastric (NP_001003209) were used to search the catalytic residues of neutral and acid lipase protein sequences, respectively. Alignments were conducted by the Clustal Omega program (www.ebi.ac.uk/Tools/msa/clustalo, accessed on 6 September 2021) [38] and edited by the software GeneDoc v2.7.0 (https://genedoc.software.informer.com/download/, accessed on 6 September 2021) [39].

### 2.6. β9 Loop and Lid Motifs

The presence and length of lids and loops in the insect neutral lipases were determined from sequence alignments with the human pancreatic lipase (HPL) (Figure S1). The β9 loop is contained between residues His204 and His224, and the lid domain is defined between residues Cys238 and Cys262 [40]. It is generally acknowledged that the β9 loop with >15 residues and the lid with at least 18 residues in length is essential for the TAG hydrolytic activity of lipases [13]. Insect acid lipases were examined for the presence of a cap domain by comparison with the human gastric lipase (HGL) (Figure S2). The cap domain occurs between residues Thr184 and Asn308 [41].

### 2.7. Prediction of Signal Peptide and Subcellular Locations

Their signal peptides were predicted by the online program SignalP-5.0 (http://www.cbs.dtu.dk/services/SignalP/, accessed on 16 September 2021) (Figure S3). The potential subcellular location was predicted by WoLF PSORT (https://wolfpsort.hgc.jp, accessed on 16 September 2021) [42] using the ProtComp v9.0 in the softberry package (http://www.softberry.com/berry.phtml, accessed on 16 September 2021).

### 2.8. Gene Expression Pattern Predicted by Codon Adaptation Index Analysis

To approximately predict the level of expression of these lipase genes, we estimated the codon adaptation index (CAI) values with a CAIcal server (http://genomes.urv.es/CAIcal/, accessed on 16 September 2021) [43]. This is a quantitative value that indicates how frequently a favored codon is used amongst highly expressed genes, referring to the coherence of coding region synonymous codons with optimal codon usage frequencies [44,45,46]. CAI analysis uses the sequence of a highly expressed gene as a reference to evaluate the degree of codon usage frequency between the target gene and the reference sequence. The CAI value is sequence-length independent, depending only on the amino acid frequency. CAI values range between 0 and 1. It is indicated that the gene is well expressed if the CAI value is higher than 0.5, while it is low expressed if the CAI value is lower than 0.03 [47]. The codon usage table of *N. vitripennis*, which is closely related to the 12 fig wasps, was used as a reference species codon usage table.

### 2.9. Protein Structure

To reveal the protein structure of five lipases in fig wasps, a Phyre2 structure prediction server (http://www.sbg.bio.ic.ac.uk/phyre2/, accessed on 20 September 2021) [48] was used to conduct alignment and tertiary structure prediction. Phyre2 was used in the alignment of hidden Markov models for homology-based protein modelling. It also incorporated the ab initio folding simulation into model regions with no detectable homology to known structures. Predicted tertiary structures and related sites were visualized by PyMOL 2.4.1 (https://www.pymol.org/2/, accessed on 20 September 2021).

### 2.10. Gene Function Prediction

Cytoscape (https://cytoscape.org, accessed on 3 September 2021) is an open-source software project for integrating biomolecular interaction networks with high-throughput expression data and other molecular states into a unified conceptual framework [49,50]. Cytoscape was used to analyze the pathways that the lipases of fig wasps involved.

## 3. Results

### 3.1. Identification of Lipases in Fig Wasps

In total, 481 lipase genes were annotated in 12 fig wasps. Except lipase2, all the other five gene families were identified. Total numbers of genes across the five lipase families in the 12 fig wasp genomes varied over more than two folds, from a low of 29 in *C. fusciceps* and *K. gibbosae* up to a high of 65 in *P. tridentata* (Table 1). The total number of lipase genes was significantly higher in NPFWs than that the PFWs (Kruskal Wallis Test, Chi-Square = 8.250, df = 1, *p* = 0.004). The family distribution across the 12 fig wasps were similar. Neutral lipases were the most abundant, followed by acid lipases (Table 1). All the genomes contained at least one HSL and lipase 3 genes. GDSL lipases range from 0 to 2 members (Table 1).

The high level of variation in lipase gene content among fig wasps was largely due to differences in their complements of neutral and acid lipases, accounting for 84.0–95.4% of the total (Table 1). The NPFWs had significantly more neutral (Kruskal Wallis Test, Chi-Square = 8.280, df = 1, *p* = 0.004) and acid (Kruskal Wallis Test, Chi-Square = 7.578, df = 1, *p* = 0.006) lipase genes than the PFWs.

### 3.2. Gene Family Expansion and Contraction

**Figure 1:**
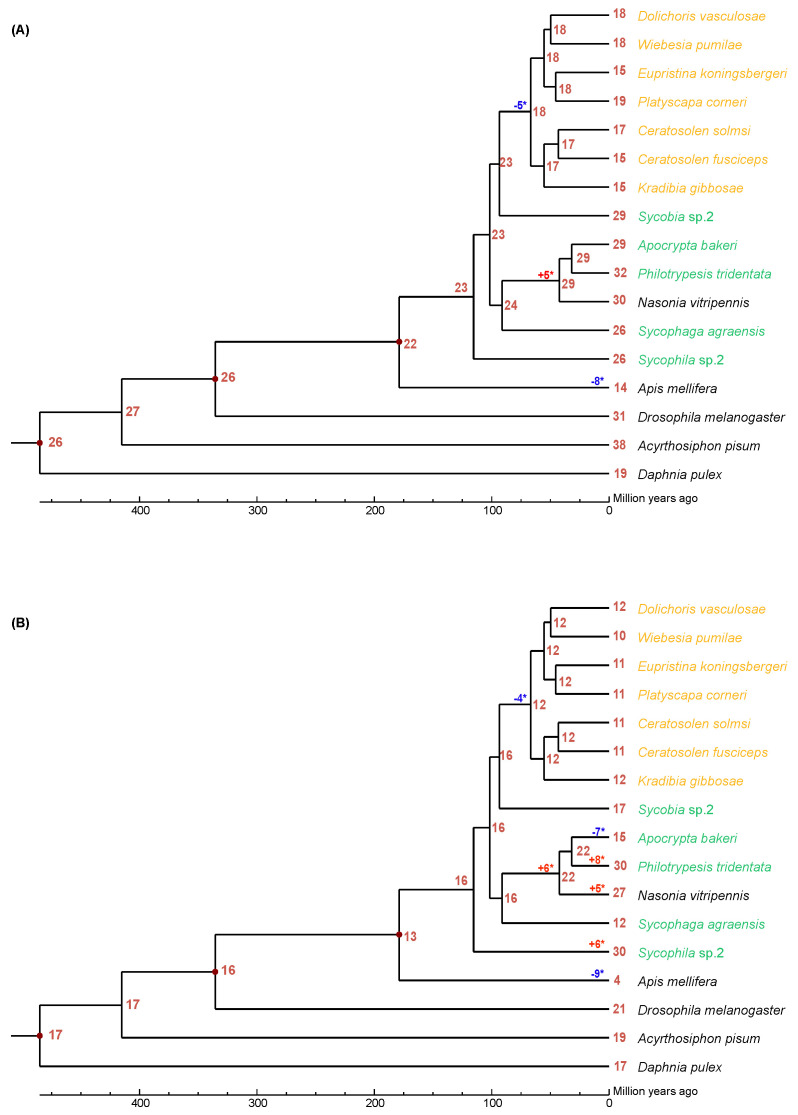
The expansion and contraction of lipase gene family. Numbers on each node are the estimated ancestral copy numbers. The names of pollinator fig wasps are indicated with orange and that of non-pollinating fig wasps are indicated with green. (**A**) neutral lipase, (**B**) acid lipase. The significant expansion and contraction of lipase gene families were indicated with asterisk (*).

We used CAFÉ analysis to estimate the expansion and contraction of neutral and acid lipase gene families in the 12 fig wasps (Figure 1). It was estimated that the most recent common ancestor of the Chalcidoidea had approximately 23 neutral lipase genes (Figure 1A). There was a net loss of five neutral lipase genes during the evolution of the PFWs from their common ancestor with *Sycobia* sp.2. There was a net gain of five neutral lipase genes during the evolution of Pteromalidae (*A. bakeri*, *P. tridentata*, *N. vitripennis*) from its common ancestor with *S. agraensis*.

It was estimated that the most recent common ancestor of the Chalcidoidea had approximately 16 acid lipase genes (Figure 1B). There was a net loss of four neutral lipase genes during the evolution of the PFWs from their common ancestor with *Sycobia* sp.2. There was a net gain of six acid lipase genes during the evolution of Pteromalidae (*A. bakeri*, *P. tridentata*, *N. vitripennis*) from its common ancestor with *S. agraensis*. Interestingly, *A. bakeri* had a net loss of seven acid lipase genes, while *P. tridentata* had a net gain of eight acid lipase genes from their common ancestor. Surprisingly there was a net gain of 14 acid lipase genes during the evolution of *Sycophila* sp.2.

### 3.3. Tandem Duplications of Lipase

**Figure 2:**
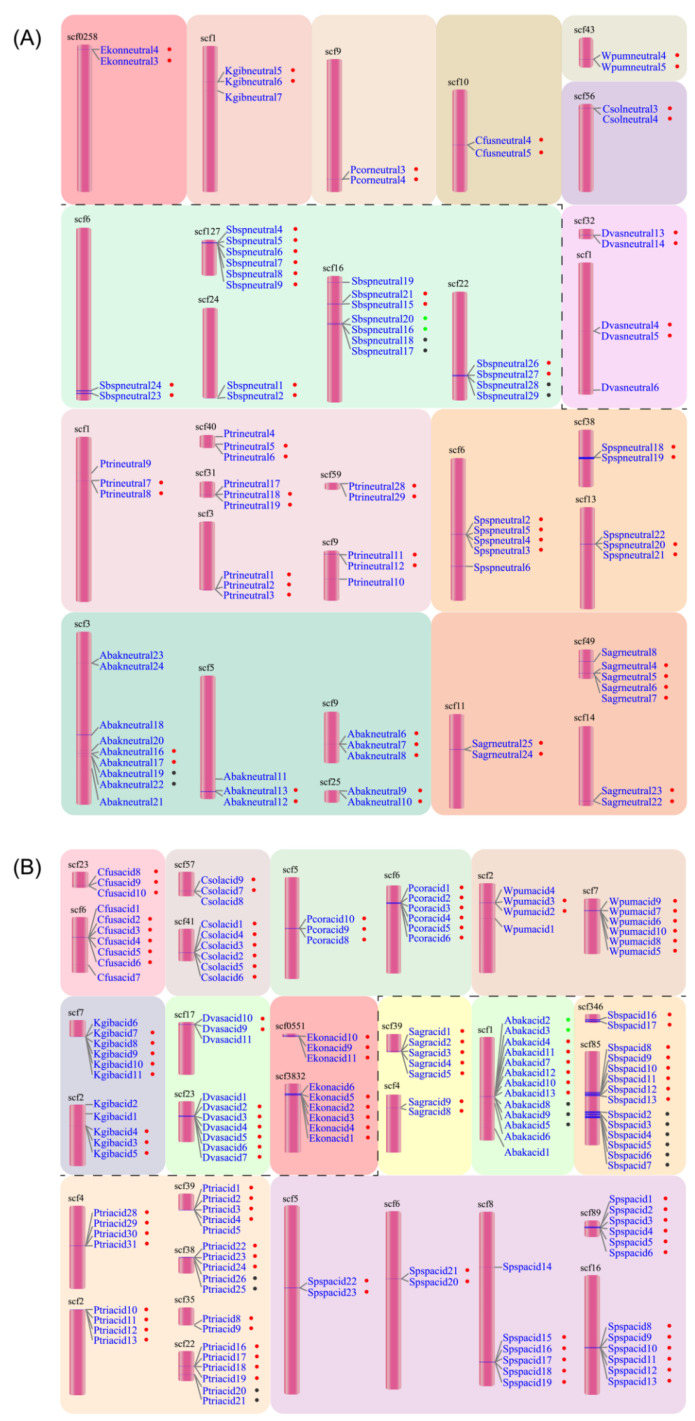
Tandem duplication events of neutral (**A**) and acid (**B**) lipases occurred in 12 fig wasp species. The pollinator fig wasps are above the black dashed line, and the non-pollinating fig wasps are below the black dashed line. All the lipase gene names are listed close to the scaffolds. The dots beside the names with the same color indicate the same tandem duplication event. The first four letters of each gene name are the abbreviation of each fig wasp species.

In the neutral lipases of the 12 fig wasps, there were 33 duplication events located in 29 scaffolds (Figure 2A). The neutral lipase gene clusters included one cluster with six members, two clusters with four members, two clusters with three members, and the other 28 smaller clusters with two members. Each PFW had only one duplication event located in one scaffold, except for *D. vasculosae*, while each NPFW had at least two duplication events; in particular, *Sycobia* sp.2 harbored eight duplication events. The tandem duplication was attributed to 10.5–40.6% of the total number of neutral lipases except for *Sycobia* sp.2, accounting for approximately 69.0%.

In the acid lipase genes, there were 35 duplication events located in 30 scaffolds (Figure 2B). The acid lipase gene clusters included nine clusters with six members, five clusters with five members, four clusters with four members, six clusters with three members and 11 clusters with two members. Each PFW had two duplication events, including eight or nine members totally. The NPFWs contained two to eight duplication events, with the number of members ranging from two to six.

Generally, the gene cluster of acid lipase harbored more members than that of neutral lipase. Moreover, the tandem duplication attributed to 58.3–83.3% of the total number of acid lipases. On average, 74.2% of the acid lipases were involved in the tandem duplication events, while this portion was 29.3% for neutral lipases.

### 3.4. Phylogenetic Analysis

**Figure 3:**
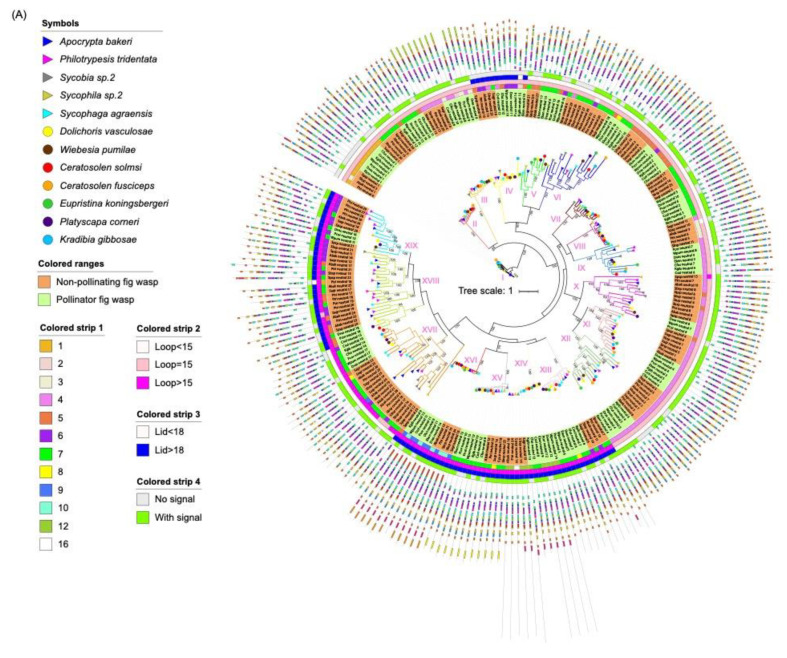

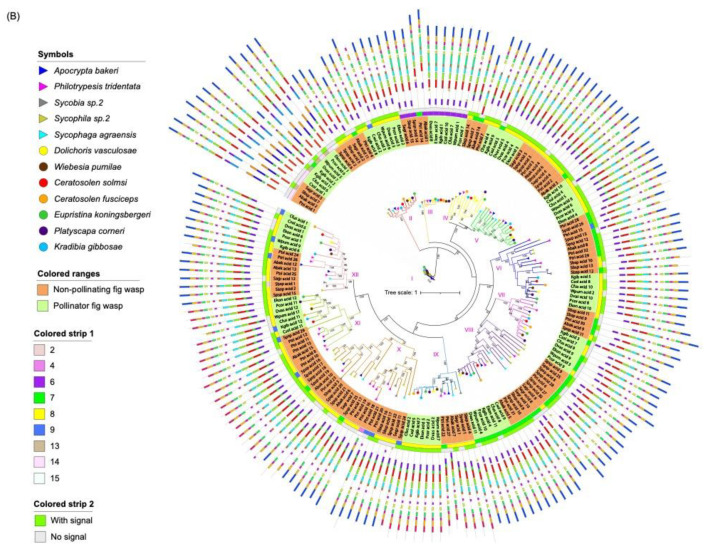
Phylogenetic tree of lipase proteins from 12 fig wasp species. (**A**) The ML tree depicts the evolutionary relationships among 259 neutral lipase protein sequences from 12 fig wasp species. From the inner to the outside: the first colored strip indicates the number of exons; the second colored strip indicates the length of the beta loop; the third colored strip indicates the length of the lid; the fourth colored strip indicates if the protein has secretion signal. (**B**) The ML tree depicts the evolutionary relationships among 182 acid lipase protein sequences from 12 fig wasp species. From the inner to the outside: the first colored strip indicates the number of exons; the second colored strip indicates if the protein has a secretion signal. The species names of fig wasps were indicated with different symbols. The background with orange indicates non-pollinating fig wasps and the background with green indicates pollinator fig wasps. The protein sequences for each motif are in supplementary Figure S5.

Two phylogenetic trees were constructed using 259 neutral lipases and 182 acid lipases predicted in 12 fig wasps, respectively (Figure 3). The neutral lipases can be divided into 19 clades (Table 2). In clade I, III, IV, VII, VIII, XIII, XIV, XV and XVI, each of the 12 fig wasps had one neutral lipase gene (Figure 3A). Clade II consisted of 11 lipase genes from 11 fig wasps, except for *E. koningsbergeri*. In clade IX, XI and XII, each of the seven pollinating fig wasps had one neutral lipase gene, while the five NPFWs had more than one neutral lipase gene. In clade X, XVIII and XIX, each of the three pollinating fig wasps had one neutral lipase gene, while the NPFWs had more than one neutral lipase gene. In clade XVII, each of the six PFWs (*E. koningsbergeri*, *P. corneri*, *K. gibbosae*, *C. fusciceps*, *C. solmsi*, *D. vasculosae*, and *W. pumilae*) had one neutral lipase gene, while each of the NPFWs had more than one neutral lipase gene. The clade V had the least 9 genes. The gene expansion of *A. bakeri* and *P. tridentata* mainly occurred in clade XVII, XVIII and XIX. Compared with lipases from other clades, clade XIII, XIV, XV and XVI clustered together, and contained an additional 3′ domain of tandemly repeated sequences of varying length and composition. The lipase of clade XIV had the longest length of introns.

The acid lipases can be divided into 12 clades (Table 2). In clade II, III, V, VI, VII, VIII, IX, XI and XII, each of the seven pollinating fig wasps had one acid lipase gene, and the NPFWs had at least one acid lipase gene (Figure 3B). In clade I, *K. gibbosae* had two genes, and the other 11 species had one acid lipase gene. Clade IV lacked an acid lipase gene from *S. agraensis* and *W. pumilae*, consisting of two genes from *D. vasculosae*, and the other fig wasp species had one acid lipase gene in this clade. Clade X only contained the acid lipases from NPFWs. The expansion of the acid lipases of *P. tridentata* and *Sycophila* sp.2 mainly occurred in clade V, VI, VIII and X. Compared with lipases from other clades, the lipases of clade I, except Kgib_acid_2, contained an additional 5′ domain of the tandemly repeated sequence.

### 3.5. Sequence Characterization

#### 3.5.1. Incomplete Catalytic Triads in Predicted Lipases

Ten of the neutral lipases did not have the consensus Ser-His-Asp triad, accounting for 3.9% of the total number (Table S2). Four of them lacked His, three of them had Gln, Asn and Ala in the position of His, two of them had Asp and Ala in the position of Ser and the last one had Glu in the position of Asp.

Five of the acid lipases had incomplete catalytic triads, accounting for 2.7% of the total number (Table S2). Two of them had Glu in place of Ser, two of them lacked Ser and the last one lacked His. The five acid lipases were only in *W. pumilae*, *Sycophila* sp.2 and *P. tridentata*, and all the other nine fig wasp species contained complete catalytic triads.

#### 3.5.2. Identification of β9 Loop and Lids of Lipases

Two neutral lipases had a β9 loop with fewer than 15 residues, 154 neutral lipases had a β9 loop with exact 15 residues and 103 neutral lipases had a β9 loop with more than 15 residues (Table 3). 93 neutral lipases had lid domains more than 18 residues, while 166 neutral lipases had a lid domain fewer than 18 residues. 81 neutral lipases have both β9 loops greater than 15 residues and lids greater than 18 residues in length. Besides that, 12 neutral lipases have lids greater than 18 residues, while the length of β9 loops were equal to 15 residues. The neutral lipases with longer loops and lids were highly clustered in clade XIII, XIV, XV, XVI, XVIII and XIX in the phylogenetic tree (Figure 3A). Among the ten neutral lipases with incomplete catalytic triads, three of them had a required β9 loop and lid length, while all of the other seven neutral lipases had a shorter lid length.

The acid lipases possess a lid or cap domain that covers the active site. All of the 182 acid lipases examined in fig wasps appeared to possess the primary sequence characteristics of the cap domain. The cap domain in human gastric acid lipase was between Thr184 and Asn308. However, Thr was replaced by Phe, Tyr, Ile and Asn, while Asn was replaced by Asp, Pro, Cyc and Lys in some acid lipases of fig wasps.

#### 3.5.3. Secretion Signals

The N-terminal sequences of the majority of the insect neutral and acid lipases conform to the consensus sequence for eukaryotic secretion signals (Figure S3). In total, 30.5% (79/259) of the neutral lipases and 37.9% (113/182) of the acid lipases lacked signal peptides (Figure 3). It was noted that 75% of the acid lipases in *S. agraensis* lacked the signal peptides, while only 18.2% of the acid lipases in *P. corneri* lacked the signal peptides. The neutral lipases lacking the signal peptide in each fig wasp species ranged from 19.2% to 46.7%. All the gene members of clade IV in the neutral lipase phylogenetic tree and clade III in the acid lipase phylogenetic tree lacked signal peptides. In other clades, the lipases without signal peptides were scattered across the tree without a consensus pattern. If the lipases without signal peptides are excluded, the number of neutral (Kruskal-Wallis test, Chi-square = 2.981, df = 1, *p* = 0.084) and acid (Kruskal-Wallis test, Chi-square = 2.753, df = 1, *p* = 0.097) lipases will have no significant difference anymore between NPFWs and PFWs. The NPFWs had more neutral lipases without signal peptides than PFWs (Kruskal-Wallis test, Chi-square = 8.134, df = 1, *p* = 0.004).

Based on the predication of subcellular locations with ProtCompt software, the majority of acid lipases were secreted extracellularly (79/113), and the other acid lipases were located in endoplasmic reticulum (6), mitochondrial (4), plasma membrane (7), and lysosomal (17). The majority of neutral lipases were secreted (116/180), and the other neutral lipases were located in lysosomal (10), Golgi (8), mitochondrial (17), plasma membrane (22) and endoplasmic reticulum (7).

### 3.6. Structure Analysis

#### 3.6.1. Exon/Intron Arrangements

**Figure 4:**
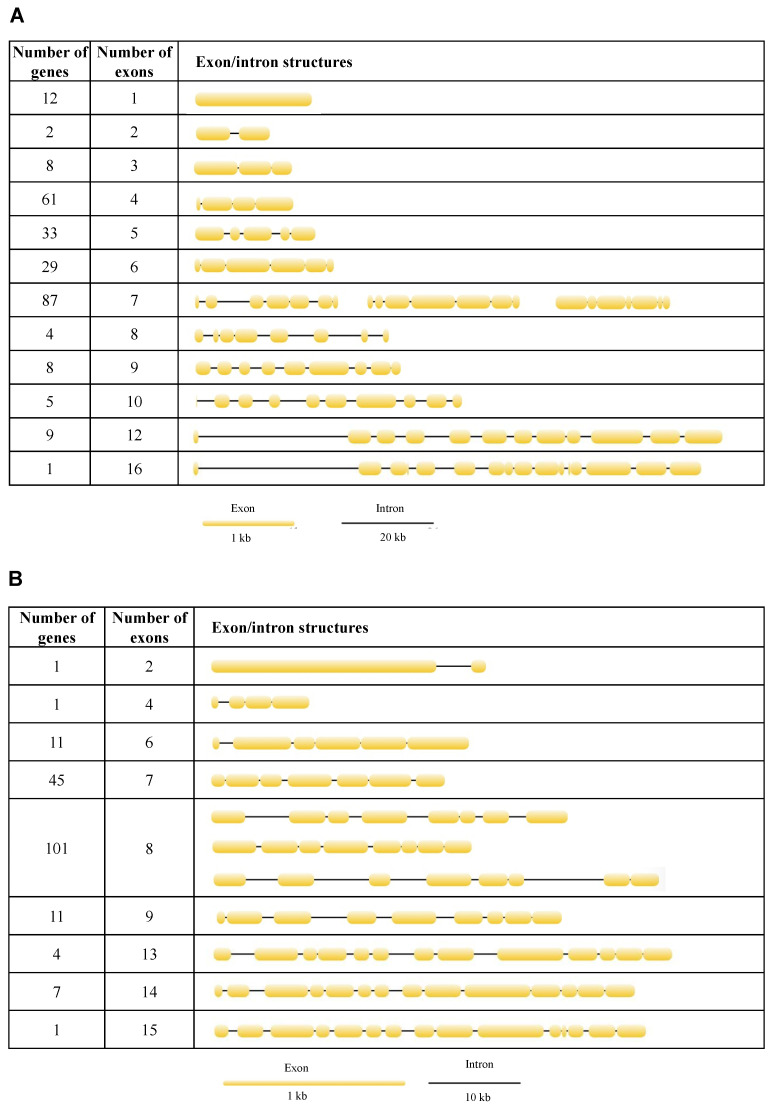
Exon/intron structures of neutral (**A**) and acid (**B**) lipases in 12 fig wasp species.

To better understand the structural diversity of lipase genes, intron/exon organizational maps were generated from the coding sequences of each lipase gene (Figure 4). The results revealed that the number of exons varied from 1 to 16 and from 2 to 15 for the neutral and acid lipase gene family, respectively (Figure 3). 23.55%, 12.74%, 11.20% and 33.59% of the neutral lipases have four, five, six and seven exons, respectively, occupying 81.08% of the total number (Figure 4A). 24.73% and 55.49% of the acid lipases have seven and eight exons, occupying 80.22% of the total number (Figure 4B). Therefore, seven exons and eight exons were predominately in neutral and acid lipases, respectively. In the phylogenetic tree, the intron/exon arrangement was similar within each clade, while it was different between different clades (Figure 3A). Phylogenetically closely related genes are structurally more similar (Figure S4). The lipases in clade I of the neutral phylogenetic tree harbored only one exon.

#### 3.6.2. Motifs

Identification of conserved protein motifs was conducted, and 20 distinct motifs were loaded (Figure S5). The motifs with catalytic triads almost remain conserved in all the proteins across 12 fig wasp species (Figure 3). The lipases from the same clade habitually exhibited a common motif structure, indicating functional resemblances with these proteins, whereas the divergences in motif compositions was obvious between different clades. It is evident that some motifs are specific to the specific clade.

#### 3.6.3. Tertiary Structures

**Figure 5:**
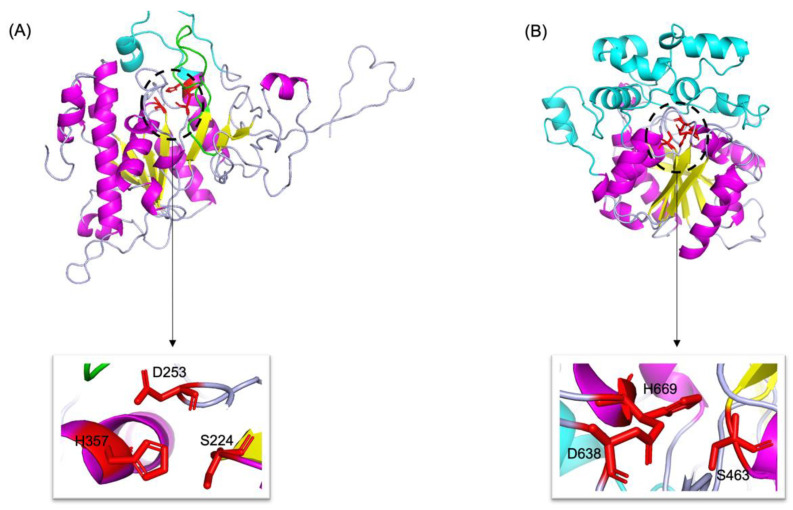
3D structures of neutral (**A**) and acid (**B**) lipase in fig wasp. α-Helices, β-sheets and loops are shown in magenta, yellow and light blue, respectively. The catalytic triads are highlighted by red sticks and zoomed in. In neutral lipase (**A**), the β9 loop was highlighted by green, and the lid was highlighted by cyan. In acid lipase (**B**), the cap domain was highlighted by cyan.

By combining multiple templates modelling and simplified ab initio folding simulation, we modelled the molecular structures of neutral (Abak_neutral_17) and acid (Abak_acid_2) lipase, using the hydrolases (PDB ID: 1hpl and 1hlg) as the templates (Figure 5). For neutral lipase (Figure 5A), a total of 345 residues have been modelled with 100% confidence, 84% coverage and 32% identity with the template. For acid lipase (Figure 5B), a total of 366 residues have been modelled with 100% confidence, 53% coverage and 39% identity with the template.

### 3.7. Expression Profiles and Function Prediction

There was no low-expression gene in GDSL (0.316–0.48), HSL (0.313–0.637) and lipase3 (0.41–0.71) (Table S3). The CAI values of 259 neutral lipase genes ranged from 0.278 to 0.823, among which 3.47% (9/259) and 25.87% (67/259) of the total neutral lipase genes had low and high expression, respectively. The CAI values of 182 acid lipase genes ranged from 0.29 to 0.68, among which 2.20% (4/182) and 10.44% (19/182) of the total acid lipase genes had low and high expression, respectively.

**Figure 6:**
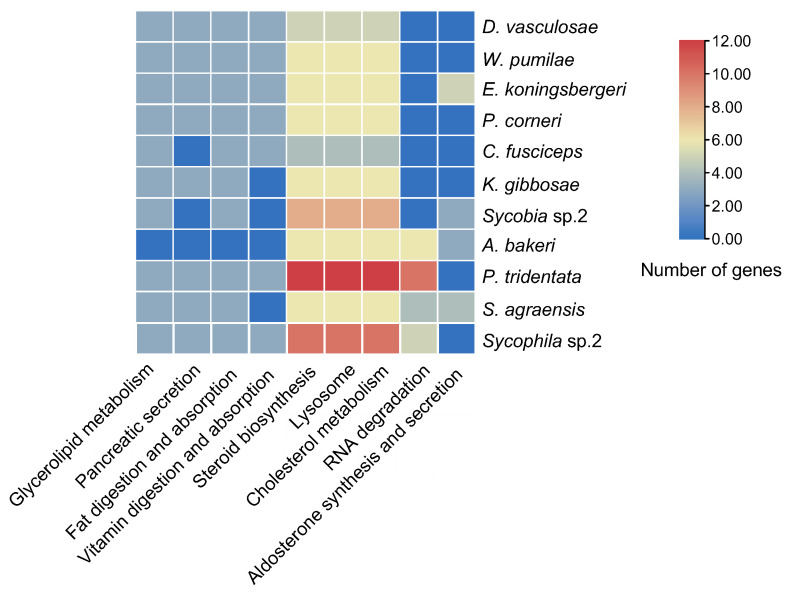
KEGG enrichment of lipase genes in 11 fig wasp species. Only data with a *p* value after Bonferroni correction of less than 0.05 are shown. The change of color ranging from orange to blue represents the number of lipase genes ranging from large to small.

Neutral lipases and GDSL were mainly involved in the pathways of steroid biosynthesis (KEGG: 00100), lysosome (KEGG: 04142), cholesterol metabolism (KEGG: 04979), glycerolipid metabolism (KEGG: 00561), pancreatic secretion (KEGG: 04972), fat digestion and absorption (KEGG: 04975) and vitamin digestion and absorption (KEGG: 04975) (Figure 6). Only the lipases in *A. bakeri*, *S. agraensis*, *Sycobia* sp.2 and *E. koningsbergeri* participated in the pathways of aldosterone synthesis and secretion (KEGG: 04925) (Figure 6). The lipases in NPFWs participated in the pathway of RNA degradation (KEGG: 03018) (Figure 6).

## 4. Discussion

In this study, we identified a total of 481 lipase genes in 12 fig wasp species belonging to neutral, acid, lipase3, GDSL and HSL lipase gene families. Lipase2 was not found in fig wasps, which was consistent with the lack of lipase2 in other insects [14]. This is the first investigation of the lipase gene family in fig wasps. Compared with two phylogenetic related *N. vitripennis* and *A. mellifera*, we can see the number of lipases in *N. vitripennis* (62) was close to that of *P. tridentata*. However, the *A. mellifera* (26) had the least number of lipases in the reported insects [14]. The expansion and contraction analysis also revealed that *A. mellifera* as well as all the PFWs had a significant contraction. The *A. mellifera* feed on pollen and nectar [51,52,53], and the PFWs feed on the endosperm tissue in the galled ovary [54]. Therefore, *A. mellifera* and the PFWs had a similar feeding regime with a rich though single food source. The contraction of the lipase gene number in PFWs and *A. mellifera* might be related to the specificity of their diet. This hypothesis definitely needs to be further tested with the lipases identified in more and more insects.

The NPFWs had significantly more lipase genes than the PFWs, especially in neutral and acid lipases. We found that the gene number of neutral and acid lipases in the clade of PFWs had significant contractions. The neutral gene family size in PFWs was approximately two-fold more than that in NPFWs. A major determinant of gene family size is gene function [55]. The acid and neutral lipases were mainly involved in lipid metabolic and catabolic process. Specifically the hydrolysis of TAG was primarily performed by acid lipases rather than neutral lipases in the reported insects [13]. More lipases participating in the lipid metabolism produce more energy for NPFWs. Indeed, the adult PFWs live for a much shorter period of time (from a few hours to 2 days) than the adult NPFWs (several days to 2 weeks) [52,53,56]. The NPFWs need more energy to maintain flight. It was found that *Panstrongylus megistus* switched fuel for sustained flight from carbohydrates to lipids [57], indicating that lipid was crucial for the continuous flight. Therefore, the flight requirements might be an important factor in driving the diversification of the lipases in NPFWs. It was also verified by the KEGG enrichment that more lipases from *Sycobia* sp.2, *P. tridentata* and *Sycophila* sp.2 were involved in the pathways of steroid biosynthesis, lysosome and cholesterol metabolism (Figure 6).

Tandem duplications are an essential source of genetic novelty, and their variation in natural populations is expected to influence adaptive walks [58]. Tandem duplication is one of the main and commonly evaluated mechanisms for gene family expansion [59,60]. Tandem gene duplication is one of the most prevalent ways of generating genes with new function [58]. We found abundant tandem duplication events in the neutral and acid lipase gene families in fig wasps. The pattern of tandem duplication between neutral and acid lipases was different. The acid lipases possessed fewer duplication events, while there were more gene members for each event. One tandem duplication event in acid lipases often resulted in more than two genes. Generally, two tandem gene copies produced a tandem duplication event in most of the neutral lipases, especially in the PFWs. It was found that tandem gene duplicates could lead to the overactivity of some genes in *Alcohol dehydrogenase*, and the expression of tandem gene duplicates was often greater than twofold [61]. Additionally, it was suggested that the relatively large number of lipase genes in the same species could be a trade-off between having sufficient catalytic diversity for rapid dietary uptake and the cost of processing DNA [62]. Therefore, the abundant tandem duplication events of lipase for fig wasps might evolve new functions, except for the lipid metabolism, which needs further studies.

None of the neutral lipase sequences in either the dipteran or lepidopteran clades meet the β9 loop and lid criteria expected of TAG lipases [13]. In fig wasps, 31.25% of the neutral lipases met the requirements of the β9 loop and lid that was concentrated in clades XIII, XIV, XV, XVI, XVIII and XIX (Figure 3A). In clade XVII, the β9 loop is longer than 15 residues, while the lid is shorter than 18 residues. Interestingly, the expansion of the neutral lipases was also predominately distributed in clade XVII and XVIII. Most insect species feed on plant tissues, of which phospholipid and galactolipid are the major lipid, rather than the TAG, which may account for the fact that most of the neutral lipases did not meet the criteria for β9 loop and lid. Additionally, the non-catalytic active lipases may fulfill other roles, such as lipid binding and storage [13].

In a previous study, we found that maltase had clear differentiations of gene structures, which led to functional divergence [63]. All the maltase genes had one or eight exons, and all the intronless genes were present in NPFWs. However, the intronless genes of lipases were only found in clade I of the phylogenetic tree of neutral lipases. The exon/intron structures of lipases were highly diverse, with a number of exons ranging from one to 16. In many eukaryotes, introns can increase gene expression without functioning as a binding site for transcription factors [64]. During the development of fig wasps, each stage requires different feeding habitats, diets and digestive enzymes. The diversity of lipases may be due to a requirement for rapid accumulation of dietary lipids.

Horne, et al. [13] found that 38% of the neutral lipases and 15% of the acid lipases lacked signal peptides. We found that it was 30.5% and 37.9% for the neutral and acid lipases of fig wasps, respectively. They were widely dispersed across the phylogenetic tree, suggesting quite ancient origins for this character and the potential conservation of function. The great majority of insect neutral and acid lipases were secreted proteins. It was consistent with their subcellular locations that the majority of lipases were predicted to be secreted extracellularly in this study.

## 5. Conclusions

In a summary, our genome-wide identification of lipase genes contributes to the understanding of digestive enzymes in fig wasps, which is essential to figure out how fig wasps utilize the lipid for their development and mobilization. The lipase gene sequences, structures and properties were characterized in this study. NPFWs possessed significantly more lipases than PFWs, which was related to their discriminative life histories and feeding regimes. The diversity of neutral and acid lipases might be due to a requirement for flight and diet specificity. Our results provided invaluable evidence from the perspective of insect metabolism to understand the obligate mutualism between figs and fig wasps. In the future, more work needs to concern the biochemical characteristics of lipases in insects.

## Figures and Tables

**Table 1:** Summary of the lipase genes in 12 fig wasps.

	Species	Abbreviation	Neutral	Acid	Lipase2	Lipase3	GDSL	HSL	Total Number
Non-pollinating fig wasp	*Apocrypta bakeri*	Abak	29	15	0	2	2	1	49
	*Philotrypesis tridentata*	Ptri	32	30	0	1	1	1	65
	*Sycobia* sp.2	Sbsp	29	17	0	2	1	1	50
	*Sycophila* sp.2	Spsp	26	30	0	1	2	1	60
	*Sycophaga agraensis*	Sagr	26	12	0	1	0	1	40
Pollinating fig wasp	*Dolichoris vasculosae*	Dvas	18	12	0	1	1	1	33
	*Wiebesia pumilae*	Wpum	18	10	0	1	1	1	31
	*Ceratosolen solmsi*	Csol	17	11	0	1	1	1	31
	*Ceratosolen fusciceps*	Cfus	15	11	0	1	1	1	29
	*Eupristina koningsbergeri*	Ekon	15	11	0	2	1	2	31
	*Platyscapa corneri*	Pcor	19	11	0	1	1	1	33
	*Kradibia gibbosae*	Kgib	15	12	0	1	0	1	29
	Total number		259	182	0	15	12	13	481

**Table 2:** The number of lipases in each clade of phylogenetic trees.

Neutral				Acid			
Clade	Number of Genes	Clade	Number of Genes	Clade	Number of Genes	Clade	Number of Genes
I	12	XI	14	I	13	XI	14
II	11	XII	16	II	12	XII	16
III	12	XIII	12	III	12		
IV	12	XIV	12	IV	11		
V	9	XV	12	V	17		
VI	16	XVI	12	VI	18		
VII	12	XVII	22	VII	12		
VIII	12	XVIII	23	VIII	21		
IX	13	XIX	11	IX	13		
X	14			X	23		

Notes: The number of clades in this table is in accordance with that in Figure 3. In the phylogenetic tree of neutral lipases in Figure 3A, there are 19 clades, and the number of neutral lipases in each clade are listed in this table. Likewise, there are 12 clades in the phylogenetic tree of acid lipases in Figure 3B, and the number of acid lipases are listed in this table.

**Table 3:** Predicted loop and lid domains of neutral lipases in 12 fig wasps. Only insect lipases with β9 loops greater than 15 and lids greater than 18 residues in length are given which have putative TAG hydrolytic activity.

Species	Gene Name	β9 Loop	Length	Lid	Length	Number
*Apocrypta bakeri*	Abak_neutral_11	HTDGSADFADGFGLLKPIGH	20	CKDVKNSVVVSHLNEDSLDINIAC	24	11
	Abak_neutral_12	HTDCSPFISGGLGISQPVAH	20	CNEGVFNSITLEKGSFFRGIKRFLGC	26	
	Abak_neutral_13	HTDGKSIFFLGLPGYGMSQPCGH	23	CTDLSETTPSLPLTLIREGLEEASRVLVAC	30	
	Abak_neutral_15	HTDGGVLGFPIPLGH	15	CELENVFAMGVGKLINRFVTC	21	
	Abak_neutral_16	HTNGRVLSRLGLGLPNPVGH	20	CILSNKASLWQYLPIPISLISETIC	25	
	Abak_neutral_17	HTNGRILRKLGLGLPYPLGH	20	CLLTKSSIWNYLPLPIESTYRSMVCFYLFIYFVLFVEIQKTIC	43	
	Abak_neutral_18	HSNGEQLILGGLGSWQPMGD	20	CSNLFVGAVSDIIWSSPVEGRSLC	24	
	Abak_neutral_23	HTDGAQERNRAFGLYDAIGH	20	CLSRRIDSQYTYEYAVKLFGHTINTRAC	28	
	Abak_neutral_24	HTDGARRRNSAFGILEPIGH	20	CQSGRRSMPTWGSVINFAFEAYQHIESNGPC	31	
	Abak_neutral_25	HTNGQYLKKLGLGLPEPIGH	20	CALTSFSIPILSIPRETVNKAIC	23	
	Abak_neutral_26	HTNARNILLLGLGLPEQLGN	20	CANIDAKFWDFLLLPINIVKSAIC	24	
*Philotrypesis tridentata*	Ptri_neutral_10	HTDGSVDFADGFGLLKPIGH	20	CNDVKNSVVVSHLNEDSLDINIAC	24	15
	Ptri_neutral_11	HTDGKSIFFLGLPGYGMSQPCGH	23	CTDLSETTPSLPLTLIREGLEEASRVLVAC	30	
	Ptri_neutral_12	HTDCSPFISGGLGISQPVAH	20	CNEGVFNSITLEKGSFFRGIKRFLGC	26	
	Ptri_neutral_13	HTDGGVLGFPIPLGH	15	CNIANVFAMGVNRIINRFITC	21	
	Ptri_neutral_17	HSNGDHFLNGGLGLIEPIGH	20	CTEVNIPVIDVKARTEPVYKAIC	23	
	Ptri_neutral_18	HANADKFLKGGLGLVEPIGH	20	CTEIKVPLPLVPLRIKTDVAFRAIC	25	
	Ptri_neutral_19	HTDTDPLQKGGLGLADPIGH	20	CSDIQLPYYLPFPIKWLEKRIAKATC	26	
	Ptri_neutral_21	HTNGGILKKLRLGLPNPSGD	20	CILMKSSLWEYLPLPIEKIESTIC	24	
	Ptri_neutral_22	HTDGAQKMNSAFGIFEPIGH	20	CGRTKRTPAIGDVIKKIFKVADHVLNHGSC	30	
	Ptri_neutral_24	HSNGEQLILGGLGSWQPMGD	20	CSNLFVGAVSDIIWSSPVEGRSLC	24	
	Ptri_neutral_26	HTDADALYKGGVSLFEPIGH	20	CSKIQLPDDLPHPIKLLKKLIERAIC	26	
	Ptri_neutral_27	HTNVKMYLNLGIGLPDRLGH	20	CKKINTAFWNFLLLPIHIVEWAIC	24	
	Ptri_neutral_28	HTNADHILRLGFGLPDRLGH	20	CEKINATFWNFLLLPIHIVQAAIC	24	
	Ptri_neutral_29	HTNAQSILLAGLGLPDRLGH	20	CEKIDVKFWDFLMLPVGVIKTAIC	24	
	Ptri_neutral_30	HTNAQNILLLGLGLPEQLGD	20	CAKINPKIWDFLLLPVDIVKSAIC	24	
*Sycobia* sp.2	Sbsp_neutral_10	HTDGSIDFTDGFGLLKPIGH	20	CRDVKNSVVVSHLNEDNLDIHIAC	24	7
	Sbsp_neutral_14	HSNGEQLILGGLGSWQPMGD	20	CSNLFLGAVSDIIWSSAVEGRSLC	24	
	Sbsp_neutral_15	HTNGRLLRQLGLGLPFPIGH	20	CVVKKSSFWKYLPLPFRKIERTLC	24	
	Sbsp_neutral_21	HTNGRGLLKLGLGLPNPLGH	20	CNITRSFIERVLPLPFKTIKETIC	24	
	Sbsp_neutral_22	HTDGGVLGFPIPLGH	15	CNIDNILAMGLNKIINRFSES	21	
	Sbsp_neutral_23	HTDCSPFISGGLGISQPVAH	20	CNEGVLNSITLEKGSFFRGIKRFLGC	26	
	Sbsp_neutral_24	HTDGKSIFFLGLPGYGMSQPCGH	23	CTDLSETTPSLPLTLIREGLEEASRVLVAC	30	
*Sycophila* sp.2	Spsp_neutral_7	HTDGSADFADGFGLLKPIGH	20	CNDVKNSVVVSHLNEDSLDIHIAC	24	11
	Spsp_neutral_11	HTNGRVLSKLGLGLPNPVGR	20	CIESESSFWKYLPMPVKKISETIC	24	
	Spsp_neutral_14	HSNGEQLILGGLGSWQPMGD	20	CSNLFVGAVSDIIWSSPVEGRSLC	24	
	Spsp_neutral_16	HTDGGVLGFPVPLGH	15	CDLGNVIAMGIGSLLNRYVTC	21	
	Spsp_neutral_18	HTDCSPFISGGLGISQPVAH	20	CNEGVFNSITLEKGSFFRGIKRFLGC	26	
	Spsp_neutral_19	HTDGKSIFFLGLPGYGMSQPCGH	23	CTDLSETTPSLPLTLIREGLEEASRVLVAC	30	
	Spsp_neutral_20	HTNARSIMLLGFGLPEDLGH	20	CDDIDASSWGSLAYPKSALTSAIC	24	
	Spsp_neutral_21	HTNAKHLFFLGLGLPNQLGY	20	CLDIDLSLWGFLMLPKDIILESIC	24	
	Spsp_neutral_22	HTNGRIWTDLGLGLPQSIGH	20	CESTDFKIPVLSIPKEAIAKMIC	23	
	Spsp_neutral_24	HTDGARTKNGAFGLIKPLGH	20	CESTNNSRRYLNYLSLFQHALGCTNC	26	
	Spsp_neutral_25	HTNSEPEKRMKDNLGTYDPLGH	22	CDLNKDARSLTYLKEIVETFLADSLRFITRTILKIDATNIILKYLNNIIPGSEERFDLIC	60	
*Sycophaga agraensis*	Sagr_neutral_9	HTDGSIDFADGFGLLKPIGH	20	CNDVKNSVVVSHLNEDSLDIHIAC	24	9
	Sagr_neutral_10	HTDGGVLGFPIPLGH	15	CNIENVLAMGFSKIINRYITC	21	
	Sagr_neutral_12	HTNARILAKLGLGLPNPIGH	20	CTLNVPLWKFLPIPLRKISEMIC	23	
	Sagr_neutral_13	HSNGEQLILGGLGSWQPMGD	20	CSNLFLGAVSDIIWSSPVEGRSLC	24	
	Sagr_neutral_15	HTDGKSIFFLGYGMSQPCGH	20	CTDLSETTPSLPLTLIREGLEEASRVLVAC	30	
	Sagr_neutral_16	HTDCSPFISGGLGISQPVAH	20	CNEGVFNSITLEKGSFFRGIKRFLGC	26	
	Sagr_neutral_17	HTNGRHLLQIGLGLPESIGH	20	CQLKNSLYIPGINLPREAIHKAVC	24	
	Sagr_neutral_18	HTNANNLLLLGLGLSEQLGN	20	CERIDASIWSFLLLPVNIIKEAIC	24	
	Sagr_neutral_19	NTNIQNSNELKSLGH	15	CDDSNFILPQGMGLAKLIEKASC	23	
*Dolichoris vasculosae*	Dvas_neutral_7	HSDGSIDFADGFGLLKPIGH	20	CNDVKNSVVVSHLNEDSLDIHIAC	24	7
	Dvas_neutral_11	HTDGGVLGFPIPLGH	15	CNLENVLAMGISKIINRYITC	21	
	Dvas_neutral_12	HSNGEQLILGGLGSWQPMGH	20	CSNLFVGAVSDIIWASPVEGRSLC	24	
	Dvas_neutral_13	HTDCSPFISGGLGISQPVAH	20	CNEGVFNSITLEKGSLFRGIKRFLGC	26	
	Dvas_neutral_14	HTDGKSIFFLGLPGYGMSQPCGH	23	CTDLSETTPSLPLTLIREGLEEASRVLVAC	30	
	Dvas_neutral_16	HTNARNILLLGLGLPDQLGK	20	CTNINTSFWNFLLLPANIIKEAIC	24	
	Dvas_neutral_18	HTDGAISENEAFGLFDSIGH	20	CGSFLSKRSVPSLLKLLLHAVKNGVC	26	
*Wiebesia pumilae*	Wpum_neutral_6	HSDGSIDFADGFGLLKPIGH	20	CNDVKNSVVVSHLNEDSLDIHIAC	24	6
	Wpum_neutral_10	HTDGGVLGFPIPLGH	15	CNLENVLAMGISKIINRYITC	21	
	Wpum_neutral_11	HTNARNILLLGLGLPDQLGQ	20	CTNINPSFWSFLLLPAKIIKEIIC	24	
	Wpum_neutral_12	HTDCSPFISGGLGINQPVAH	20	CNEGVFNSITLEKGSLFRGIKRFLGC	26	
	Wpum_neutral_13	HTDGKSIFFLGYGMSQPCGH	20	CTDLSETTPSLPLTLIREGLEEASRVLVAC	30	
	Wpum_neutral_14	HSNGEQLILGGLGSWQPMGH	20	CSNLFVGAVSDIIWSNPVEGRSLC	24	
*Ceratosolen solmsi*	Csol_neutral_7	HSDGSIDFADGFGLLKPIGH	20	CNDVKNSVVVSHLNEDSLDIHIAC	24	
	Csol_neutral_9	HSNGEQLILGGLGSWQPMGD	20	CSNLFVGAVSDIIWSSPVEGRSLC	24	6
	Csol_neutral_11	HTDGKSIFFLGLPGYGMSQPCGH	23	CTDLSETTPSLPLTLIREGLEEASRVLVAC	30	
	Csol_neutral_12	HTDCSPFISGGLGISQPVAH	20	CNEGVFNSITLEKGSLFRGIKRFLGC	26	
	Csol_neutral_13	HTDGGVLGFPIPLGH	15	CNLENRESTICSSHVSVTC	19	
	Csol_neutral_15	HTDGALSQNEAFGLYDPIGH	20	CGFLASQKSVPSLLKLLVHAMENGIC	26	
*Ceratosolen fusciceps*	Cfus_neutral_6	HTDGSVDFADGFGLLKPIGH	20	CKDVKNSVVVSHLNEDSLDIEIAC	24	5
	Cfus_neutral_8	HTDCSPFISGGLGINQPVAH	20	CNEGVFNSITLEKGSLFRGIKRFLGC	26	
	Cfus_neutral_9	HTDGKSIFFLGLPGYGMSQPCGH	23	CTDLSETTPSLPLTLIREGLEEASRVLVAC	30	
	Cfus_neutral_12	HTDGGVLGFPIPLGH	15	CNLENVLAMGIGKIINRYITC	21	
	Cfus_neutral_13	HTNGEQLILGGLGSWQPMGD	20	CSNLFVGAVSDIIWSSPVEGRSLC	24	
*Eupristina koningsbergeri*	Ekon_neutral_5	HSDGSVDFSDGFGLLKPMGH	20	CNDVKNSVVVSHLNENSLDIHIAC	24	5
	Ekon_neutral_9	HSNGEQLILGGLGSWQPMGH	20	CSNLFVGAVSDIIWSNPVEGRSLC	24	
	Ekon_neutral_10	HTDGGVLGFPIPLGH	15	CDFENVLSMGISKIINRYITC	21	
	Ekon_neutral_11	HTDGKSIFFLGLPGYGMSQPCGH	23	CTDLSETTPSLPLTLIREGLEEASRVLVAC	30	
	Ekon_neutral_12	HTDCSPFISGGLGISQAVAH	20	CNEGVFNSITLEKGSFFRGIKRFLGC	26	
*Platyscapa corneri*	Pcor_neutral_5	HSDGSIDFADGFGLLKPIGH	20	CNDVKNSVVVSHLNEDSLDIQIAC	24	7
	Pcor_neutral_10	HTDCSPFISGGLGISQPVAH	20	CNEGVFNSITLEKGSLFRGIKRFLGC	26	
	Pcor_neutral_11	HTDGKSIFFLGLPGYGMSQPCGH	23	CTDLSETTPSLPLTLIREGLEEASRVLVAC	30	
	Pcor_neutral_12	HSNGEQLILGGLGSWQPMGH	20	CSNLFVGAVSDIIWSSPVEGRSLC	24	
	Pcor_neutral_13	HTNARNILLLGLGLPEKLGM	20	CKNIDVSFWNFLLLPAKIIKEIIC	24	
	Pcor_neutral_14	HTDGGVLGFPIPLGH	15	CNFENVLAMGISKIINRYITC	21	
	Pcor_neutral_18	HTDGAISEDEAFGLLEPIGH	20	CSLLRRRKRSAESLLKLLVHAIENGAC	27	
*Kradibia gibbosae*	Kgib_neutral_8	HSDGSVDFADGFGLLKPIGH	20	CNDVKNSVVVSHLNEDSLDIHIAC	24	4
	Kgib_neutral_9	HTDGKSIFFLGLPGYGMSQPCGH	23	CTDFGEATPLTLIREGLEEASRVLVAC	27	
	Kgib_neutral_10	HTDCSPFISGGLGISQPVAH	20	CNEGVFNSITLEKGSLFRGIKRFLGC	26	
	Kgib_neutral_13	HSNGEQLILGGLGSWQQMGD	20	CSNLFLGAVSDIIWSSSVEGRSLC	24	

## Data Availability

The data presented in this study are available in article or Supplementary Materials.

## Supplementary Materials

The following are available online at https://www.mdpi.com/article/10.3390/insects13050407/s1, Figure S1: The catalytic triad, lid and loop in neutral lipase taking *Ceratosolen fuscicepus* as an example. The catalytic triad S-D-H was circled by red rectangle, the β loop was circled by green rectangle, and the lid domain was circled by blue rectangle. Figure S2: The catalytic triad cap domain in acid lipase taking *Ceratosolen fuscicepus* as an example. The catalytic triad S-D-H were circled by red rectangle, and the cap domain was circled by blue rectangle. The motifs with catalytic triad were circled by red rectangles. Figure S3: The predicted signal sequence in lipase with Signal P software taking *Apocrypta bakeri* as an example. (A) An acid lipase with secretion signal, (B) An acid lipase without secretion signal. Figure S4: The exon-intron arrangement in all neutral (A) and acid (B) lipases. Figure S5: The sequence of 20 motifs used in the phylogenetic tree of neutral lipases (A) and acid lipases (B). Table S1: Information of 12 fig wasp samples. Table S2: The gene list of neutral and acid lipases with incomplete neutral lipase triad. Table S3: The CAI values of all the lipases in fig wasps.
