# Supplementary material for: Genome-Wide Identification and Analysis of Lipases in Fig Wasps (Chalcidoidea, Hymenoptera)
Source: Insects. 2022 Apr 24;13(5):407. doi: 10.3390/insects13050407 (PMC9143690; doi:10.3390/insects13050407)
Supplement: Supplementary file 1 [file insects-13-00407-s001.zip › Supplementary figures-final.pdf]

Supplementary data

Genome-wide identification and analysis of lipases in fig wasps (Chalcidoidea, Hymenoptera)

Xianqin Wei [a], Jiaxing Li [a], Tao Wang [a], Jinhua Xiao [a, *], Dawei Huang [a, *]

[a] Institute of Entomology, College of Life Sciences, Nankai University, Tianjin 300071, China.

[*] Corresponding authors. E-mail addresses: xiaojh@nankai.edu.cn (J. Xiao); huangdw@nankai.edu.cn (D. Huang)

```
                 *        20         *        40         *        60         *        80         *        100
Cfus_neutral_1 : ------------------------------------MKISSKRNMNGHQEINVQILQLISLFS--------------ILSQFVLIHSFDIQNSIEENELVLTKDFF :  56
Cfus_neutral_2 : -----------------------------------MTYYVHRVVILSLFI--LAGKLIIIDS---------------------KEVSPHNSLLN------EVETMI :  42
Cfus_neutral_3 : -----------------------------MVSLFTDTFFWLSWLIWGACACWESG----LKEKYDGYG------------EDWIFMPDGKGQPQV----AVIKAPY :  57
Cfus_neutral_4 : ----------------------------------MSTHAATLLILFIANAFGSIESLDVS------------------AQDLDLLNQELSVFDDDGQLVTISNEM :  53
Cfus_neutral_5 : -------------------------------MGPQNIIITIILQLVLRVGISGCQPLSMISCA---------------IGRSAELLADDEAAAGEAPSAYNTNVDV :  60
Cfus_neutral_6 : MKCEGSISVRKKCSLRFAERGQTGPICFLTGGRQAASPESNKRSDSGAEALTMYNKLLLVVLFSIPLCAGGARNNARILEKRNRIMSMHRRLSNRDEVVVGSRRIC : 106
Cfus_neutral_7 : -----------------------------------MLLFAVLISFYSIATGDIVGDLSLRQQFQSSLP------------DFLKEEAILNSYVDSLPMPPEDHFFL :  59
Cfus_neutral_8 : ----------------------------MWLKEMLLLVLICTPDLLDGSYLTNPRRLHWHSALGSGPFMPLTTQPPIEVTAFATKIAQAVEDSKLEYDEEWFMWRC :  78
Cfus_neutral_9 : -------------------------MYASQLRLECSAIRMRRRSTWKETNLESEGALVLFLIALAPAICSAG------ILDPWQWARSDRIEVNIPWLPFENETRC :  75
Cfus_neutral_10: --------------------------------MALKRETASRLTLLALIQLSVG-----------------------LFRDCEARGPIQEAIENGAAADYNKDDC :  50
Cfus_neutral_11: ---------------------------------------------------------------------------MFLMQGRVHYQLPCQAGHFIYKNDIF :  26
Cfus_neutral_12: ----------------------------------------------------------------------------MLGDSPANFSLFPTGQCLLCCP--- :  22
Cfus_neutral_13: -----------------------------MIDKLLLGLWIAHLICWNVDNAEAADDLRLIDESLDERNDAEIFRAVVQSMSEWESRKHKRGHRSK----REVSRVC :  73
Cfus_neutral_14: --------------------------------MKFSLVFSVLLILFEKSFANIN-------------------------ETMKISCLELKNIPLVGKVITCSSLA :  48
Cfus_neutral_15: -----------------------------------MYGFKWIVDVLAMIFMG-----------------------------TLICSSEKLDRSKLSSDYLNKINI :  41
NP_572286.1:      ---------------------------------MYVANTTGGLMLQTMLYLAN-----HTSRAAVNTLVDLPPAPKSDIN----------------------EVKC :  46

              *       120         *       140         *       160         *       180         *       200         *
Cfus_neutral_1 : LGPC-----------LVNTN----QTCPDQEIGFFLYTHKNEIVGQKIFVNNSGSNLDNTSFNSQDPTKIIHGYNSDMQLDSLVDIRQEYLKRNS---YNLIAVD : 144
Cfus_neutral_2 : NTAIKLIHEP---ATFKLYT----RENPFGEEQLLLNNT---------------EILYASHFNESRPTKLIIHGFSDTGKEVWIRGLIDAYLKYQD---VNVIVVG : 123
Cfus_neutral_3 : PDGRGILDET--INFILFTR----SNP-DEGLYLFTNNS---------------DILKTSYFSPLKTTKFITHGWKSNAFTESVLSLKTEFLKYGD---FNVLLVN : 138
Cfus_neutral_4 : PNELETMLIS-------------MDSHVHFFLFTRETGDNYELLTIGDVEHLRN----TSFDPARETKFVTHGWVNSGKSKCCILQREAYLMYGD---YNVIVID : 138
Cfus_neutral_5 : EKYVFFYLYT-------------KENPEGQAINLNEIHEAEEEAEEEAEEEKRQRKQLVNFNGKRETKILTHGYFSSKDAKSCKIIRDAFLRYGD---YNVIVVD : 149
Cfus_neutral_6 : FDMLGCFANRQQPLALKRPP----QDPSIINTRFHLYTRGNREEPEMIEYGDRLKSMNQSNFDGKKKLKVIHGYKGSGNNEGAVAGAHSLLDLED---ANVIVVD : 205
Cfus_neutral_7 : TSVLDPIMVPERDVSLYLFT----KSKPQEPVTLKVGDT---------------ASLRNLNLATNDSTKVIHGWTDNGNSSWIQDMRRNYLATEC---CNVIIVD : 143
Cfus_neutral_8 : YPPFGCFYIGSPWSGGSRPVSTFPVRPESINPLFMLYTRQGIEDPHQLIIDN-ISTIKNSPLRKNENLYFIHGYLDNGNKTWVLRMMSELLLRED---ANVVVVN : 180
Cfus_neutral_9 : YDELGCLNITRSWYHLIHRPLNVFPLPREVINTRFILYTNDNPTEGQVLIVGKDKSIKRSNFSPKRKTKFIHGFIDTPLSNWVKEMRSELLRHGN---YNVIVVD : 178
Cfus_neutral_10: TWRRGNDRDS----------------CPDPDVHIHLFTPGN---PRRKLDPSHVADWLRQDYEPTKDNVILIHGYAGGEDALPMSVLRDAYLKNGS---YNVFLVD : 134
Cfus_neutral_11: R------------------------DTQQNPLLLDVTR-------------KQSLFKSRFDRSHPTKIIHGFGGGRNLAPSTDLRKAYFTRGN---YNIIVD :  90
Cfus_neutral_12: -------------------------VNLDRDILYQLYTRRNPTIPQTIQPGN-SCSLRRSNFNASQPTVLYIHGYSERSPGISGSSIKNAYLFRNN---FNVILVD :  99
Cfus_neutral_13: YEDVGCFEDTGPFSYLEMLP----SPPKDVGTRFFVYGSRKARSIPMEVPAEDISEKTTNAIDPELPTKVIVHGFGSSCDHIWVYEMRSALMAVIE---CNIVCVD : 172
Cfus_neutral_14: S------------------------GNDQLVRYYLSTNN---IRTPKEFNPCDLSKIQSHFDPLEETIIIHGYLASVKRDWILDLKDKLLDAKK---GNVIMID : 123
Cfus_neutral_15: VHYQG--------------------STAKNANSYKYPIN------------KATELKNNLDMKKPFTLYVHGYSENYTEESVQTVLDAHLVKGD---DNFVILD : 110
NP_572286.1:      FGVYGCFPINGPWNTVTRSINVHPQKPSEIEPHFTLHTRRALDQPKYLDLND-PESVQGMGMNPKGKIFLLVHGYLESGEIPWMWDMAKALLAHEPEGRASVVLID : 151

      220         *       240         *       260         *       280         *       300         *       3
Cfus_neutral_1 : WSRLASGPC-----YPIAVYNVPHVGQCLAQLIDRLKDYG-----ATDIHIIGFSLGAHVPAFAANYLRPY---KLPRITGLDPAMPLF---ITVNK-NHKLDEGD : 233
Cfus_neutral_2 : WGIL-AADP-----YPTAANNTRRVGEYLGVFLEFLCRES--NLEYKDVHMCGHSLGSHVAGFAGAFLDG----RVGRITGLDPASPLFETASGVVDPDFRLDPTD : 217
Cfus_neutral_3 : WEPMAASTF-----YLGPMRNTYKVGRKTAEFIDFLVRDT--GLATDNIHFIGHSLGAHVAGNTGELLMSG---KLGRVTGLDPALPGFHLLTADT---GRLDATD : 231
Cfus_neutral_4 : WGKIAKNP------YIWAATHVVNVGKYVAKMLDFLIDEG---IDLSTTTLSGHSLGAHIVGLAGYYAKGK----PNYIVGLDPALPLFSFAGVGS----RISTQD : 227
Cfus_neutral_5 : WWPLQSIWGPIPTLYWTVASHVNAVGKYVGKLIDYLANHG---MDLTTTSLVGHSLGAHVMGIASYQARDK----VNYIVGLDPALPLFENRNDSE----RLSADD : 244
Cfus_neutral_6 : WAKGAG-TT-----YGLAVSNTELVGRQLSLVLLDAIELG---VSSADVHLVGFSLGAHVAGCASEMLKKRG-ILVGRITGLDPASPFFRHHLFREK-TRKLDSSD : 300
Cfus_neutral_7 : WSAG-SSKE-----YLVSASLTKQVGKYVSRLLAFLLAEG--VLAYENVHVLGHSLGAHVAGTIGESLSG----RIARITGMDPARPDFEAP-VLKELGDRLDLTD : 236
Cfus_neutral_8 : WIGGASPP------YTQAVANTRLVGAMTGRLASQLIQKGN-ILPTR-LHCIGHSLGAHTCGYIGYNLRVQYGYKLSRISGLDPAEPHFSNTSPMVR----LDPTD : 274
Cfus_neutral_9 : WAGG-SLPL-----YTQATANTRLVGLEIAHLVKHLQTNY--GVELSDVHLIGHSLGAHTAGYAGEKLNG----NIGRITGLDPAEPYFQGMPSH----LRLDYTD : 268
Cfus_neutral_10: WGKLCAAPC-----YPAAVSNMRPVARCLAASLTSLRNHG---LTIERTHCVGHSLGAHVCGIMANYLLFR----MSRIVALDPARPLIRPGLVN-----RLDSGD : 223
Cfus_neutral_11: YGSLVREPC-----LSQINWGPDFCSRCIAQFVRYLRDHPR-GTPVERIHVLGYSVGAHIAGLISNYLPDD---KLGRITGLDPTIFFY---MNGNR-SRDLDETD : 183
Cfus_neutral_12: WSKLATMPW-----YVKAVGNTRIVGPHVANTLRWLESIG--AFNLRTVHVVGFSLGAEVAGFMGKALFPQ---RVGRITGLDAAYPLY---MNTGA-EGHLTAAD : 191
Cfus_neutral_13: WGPGSAVPN-----YVRAAANTRLVGRQLAK----LVRSL--NVSLEMVHLIGFSLGAHVAGFAGAELGN-----VSRITGLDPAGPLFEAQDLR----ARLDKTD : 258
Cfus_neutral_14: WSSLSTKKN-----YVHAAQNTVAATQQIHKMESSNFKASKTKQKWNKIYFIGHSLGAQVSGQTAHLLNQDPFWKVTRITGLDPAKPCFTDVEPHLR----VDKQD : 220
Cfus_neutral_15: WSAFGFDN------YIIVAQRAQKIAMHLAATIEDLVTAK--LIDIDRFRIVSHSLGSQTSGYTGQYLSFE----IPRITALDPAMPLFYGITNKH-----INKDS : 199
NP_572286.1:      WGGGASPP-----YVQAVANIRLVGAITAHVVHMLYEELR-LPNLDNVHIIGHSLGAHLSGYAGYHLQHDFGLKPARITGLDPAAPLFTDTDPIVR----LDKTD : 246

     20         *       340         *       360         *       380         *       400         *       420
Cfus_neutral_1 : AEFVDVFHTN--------AFIQGKVEASGHIDFYINGGI-NQPGCWEK-------------------RNPFGCNHHRAAEYFAESIN-SKDG---FWGWRCSG-F : 305
Cfus_neutral_2 : AQFVDVIHTSGT--------AFGFLAPLGHVDFYPNSGKFPQPGCN--------FAPTN------------TYCSHTRAYQLMTESIG-STSG---FKSKNCES-W : 290
Cfus_neutral_3 : ALFVDIIHSCG--------GILGYMKPLGHVDFYPNAGVAVQPGCC-----CVPEIIEA--------------CSHGRAYKYFTESIN-SIIG---FMAKKCEN-W : 305
Cfus_neutral_4 : AKQVEIIHTNAG--------LLGFLSPIGDADYYPNGGT-RQKGCSVDL--------------------AGACSHSRSYEFFAESIV-SDIG---FYAMNCKD-Y : 298
Cfus_neutral_5 : ANFVEVIHTDEG--------NCGMFVPVGDYDFYPNGGV-KQPGCSTN-----------------------TCSHTRSCELYAESLI-NKKG---FFAAKCSS-L : 312
Cfus_neutral_6 : ARLVDVIHTDGS---VDFADGFGLLKPIGHVDFFPNGGR-QQPGCKDVKNSVVVSHLNEDS------LDIEIACSHARSWFLFVESMR-SQLGGCRFLSWPCKRRF : 395
Cfus_neutral_7 : AQFVDVIHTCAG--------TAGFIRPIGHVDFYPNGGSFRQPGCP--------VLLT-------------QHCSHARSHQYMAESII-NPMG---FAALACNG-W : 308
Cfus_neutral_8 : ADFVTAIHTDCS---PFISGGLGINQPVAHIDFYPNGGR-NQPGCNEGVFNSITLEKGSLFRG----IKRFLGCNHIRSYEYFIESIN-AACP---FLSVPCTS-W : 367
Cfus_neutral_9 : AQLVDVIHTDGKSIFFLGLPGYGMSQPCGHLDFYPNNGK-EQPGCTDLSETTPSLPLTLIREGLEEASRVLVAGNHVRAIKLFIESIN-GKCQ---YVAHECGS-Y : 368
Cfus_neutral_10: ADFVEVIHTNAG--------YYGELGKVGHVDFCVNGGK-LQPFCENRTN-------------------EQLCSHVWAVCYMAQSVD-GNRE---MVAEPCSR-- : 294
Cfus_neutral_11: AHFVDVIHTG--------AGILGQWGPNGHADFYVNGGS-SQPGCAMSS------------------ILQTLSCDHTKVTPYYIESIT-TKVG---FWAAPCAN-L : 257
Cfus_neutral_12: AEFVDLIHTD--------GGVLGFPIPLGHADFYPNGGRPLQPGCNLEN---VLAMGIGKI------INRYITCSHHRAWRFYAESIT-DPSG---FPASRCPKWR : 276
Cfus_neutral_13: AQFVDVIHTNGE---QLILGGLGSWQPMGDVDFYPNGGR-MQTGCSNLFVGAVSDIIWSSPV------EGRSLCNHRRAYKLFTDSIS-PKCR---FPAFPCESGY : 350
Cfus_neutral_14: ADFVDIIHTQVG--YGGSIDAFGLKKSIGHVDFYVNAGIVQPPCFCTVFTT-------------EWSRMICSHRIAYKYFAESLVNSISGECKYYSYEWNGSY : 308
Cfus_neutral_15: AKIVDVIHTDAG--------IYGASSNTGTIDFYANGGTRPQPGCPITG-----------------------FCSHHRSWKFYAESVG-NADA---FQGLECPS-N : 269
NP_572286.1:      AHFVDIVHTDAN---PLMKGGLGINMRLGHVDFFPNGGF-DNPGCNKKFQD--VVKKKTLFLT----MQEFLGCNHIRSQQYFTESIG-SQCP---FLGITCDS-F : 337

            β loop              lid
          *       440         *       460         *       480         *       500         *       520         *
Cfus_neutral_1 : LTYLFGLC-PQKL----PAVLAGDPVNQTNR---------------GFFLVKTRDKSPFAEGKFTSRMFYNLNL------------------------------ : 359
Cfus_neutral_2 : EKYKDGHCDHN------PIVLMGEYASTSLR----------------GNFYLTTKYASPFAID------------------------------------------ : 331
Cfus_neutral_3 : EKYLRGECDNCQT------AFLGEHVDKSVN----------------GSFFLKTRSEPPYACIN-------------------------------------EIDDN : 352
Cfus_neutral_4 : SSFVHGTCTGEVS-------LMGGFKESLPSN---------------GTYYLGTRGKYPYAKGRN---------------------------------------- : 341
Cfus_neutral_5 : EDMRRGYCFGDVV-------LMGGDLEGPGNQ---------------QGSVLPRDE--------------------------------------------------- : 346
Cfus_neutral_6 : GSFAAGSCFPQEGT--IAAPEMGYAADQGPT----------------GLYYLSTRAQPPYCGYPLRAAVKLSEKEFRSTGLLWMRLVKGDAEVTFGIVCDVANLPP : 483
Cfus_neutral_7 : TNYKYQECDGDKD----RVAYMGEYVDKDAR----------------GIYYLETNADSPYGRGPITIT-------------------------------------- : 356
Cfus_neutral_8 : EKFQNGSCFDCVEQ---HCPRFGFHAQ---------------PGNHHASTYLMTGREKPYCKSHYRVTIVISKTMESLSYGGEVGMFFIRVFGSNGK--------T : 447
Cfus_neutral_9 : ASFLRGECFSCKSNNSLSCGIMGYHADSSPALVRRQQMGQDMSALLGSKFFFSTGKDDPYCRRHYRITINLARPPTAESWVQGYMKVTLHADNGVIRNMDLTPSGY : 474
Cfus_neutral_10: -RCPSGPRIAPRSG---ELLIMGQHTPKGSR----------------GSFCINS-YEPPYCPKYHEGRGDERCCI------------------------------ : 348
Cfus_neutral_11: FSYLIGWCNPSEE----EHVPMGEDTPHTAR----------------GIFYLSTNAHKPYARGLPVKKQRTTSPKRSCCHQY----------------------- : 319
Cfus_neutral_12: PDIRVHCRWTPDA-------FMGYAASGRLR----------------GMYYLRTNANPPYARNITGYC-------------------------------------- : 321
Cfus_neutral_13: DGLLKGECFPCRSESEVGCGLMGFYSDASQAR---------------GQLYLVTRDEEPFCAHQYMVKVYNSRHERPVKSYGKLQVTLLAEGSVNETFTMTKKDDE : 441
Cfus_neutral_14: DEAVKSLNRQRIGSYCLDCPEMGINAPQSKKT---------------GDFLVITSTTGPYCKFEEQDTKIVLKVLSRLKSVTTSTSAPNTTLEPQKTST------- : 392
Cfus_neutral_15: KDFKNNKCDGNKV------VYFGYHMPLDAT----------------GTYYFSTNDKSPFGRYANGTKA------------------------------------- : 316
NP_572286.1:      ESFKDTKCTSCEEPG-HTCLRMGYHSQEDYQE--QVDLGQLQQGDSPGVFYLWTGDSKPFCRLHYRITVRVSGHDESTLHGGEVGILSIQLHDALVKKAGKNERAA : 440

         540         *       560         *       580         *       600         *       620         *
```

Figure S1. The catalytic triad, lid and loop in neutral lipase taking *Ceratosolen fuscicepus* as an example. The catalytic triad S-D-H was circled by red rectangle, the β loop was circled by green rectangle, and the lid domain was circled by blue rectangle.

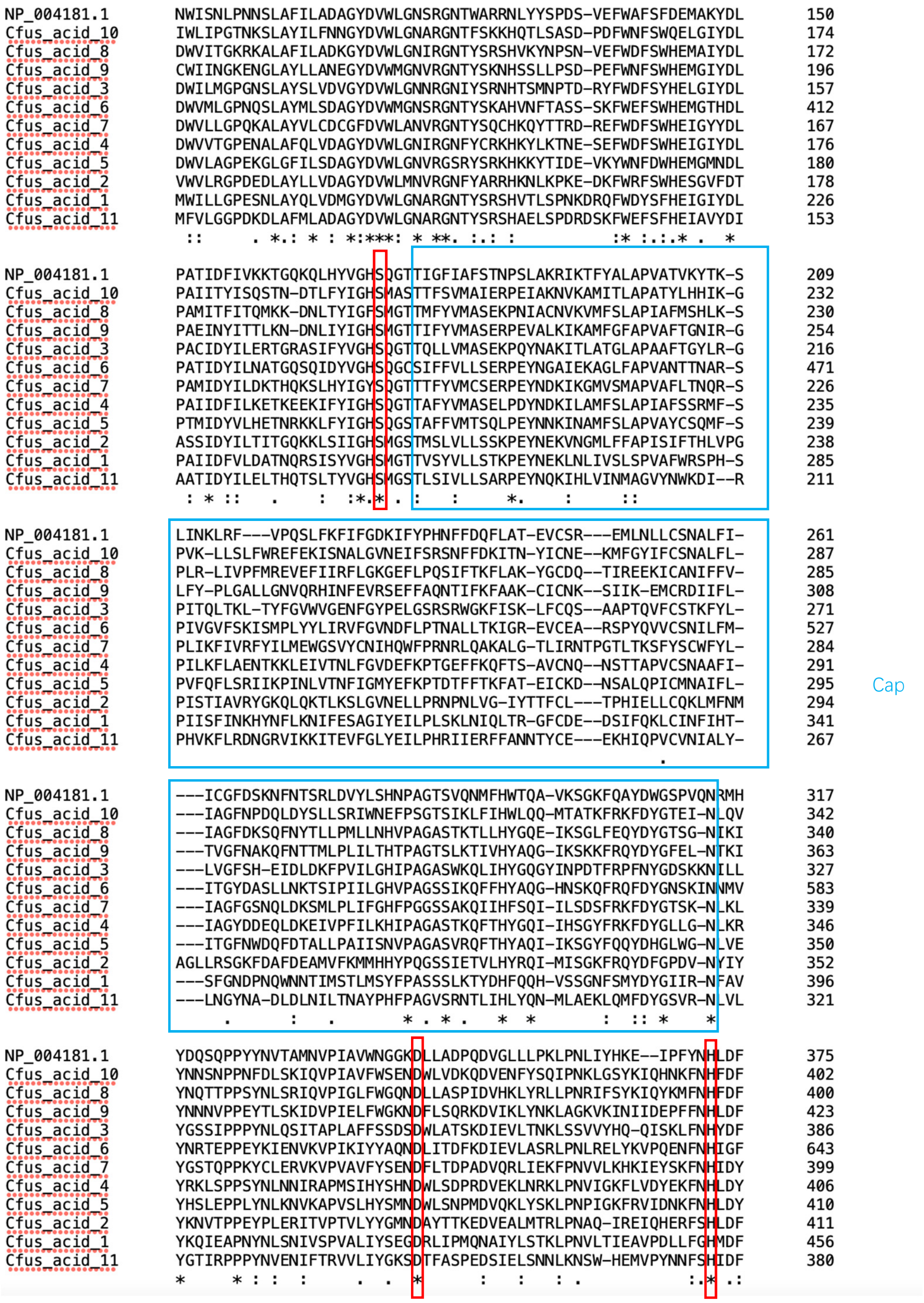

Figure S2. The catalytic triad cap domain in acid lipase taking *Ceratosolen fuscicepus* as an example. The catalytic triad S-D-H were circled by red rectangle, and the cap domain was circled by blue rectangle.

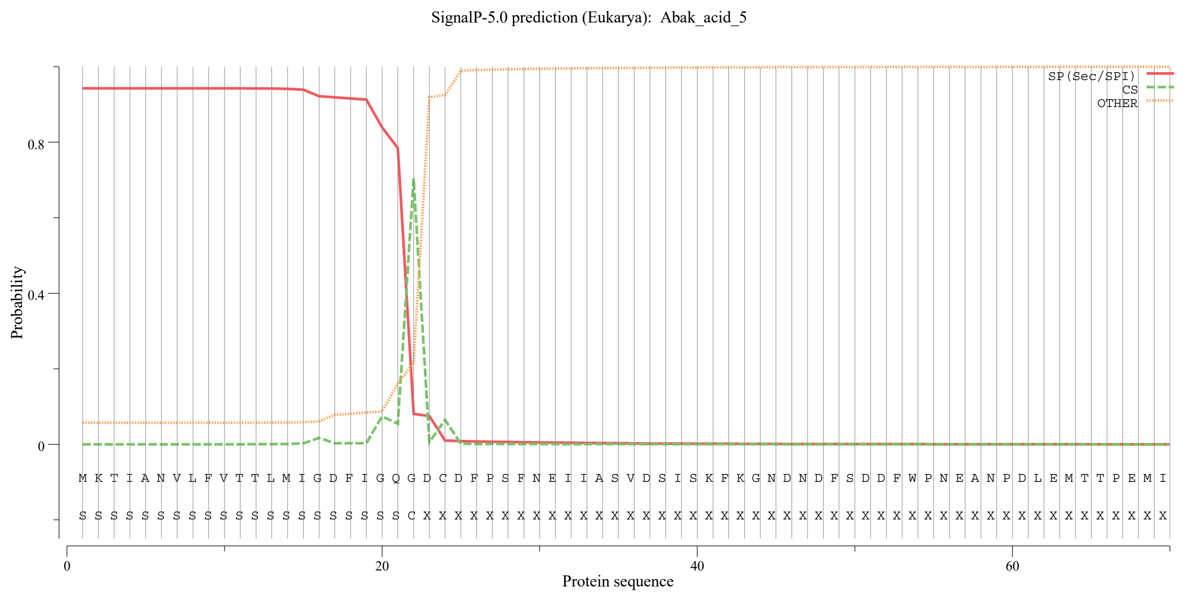

(A)

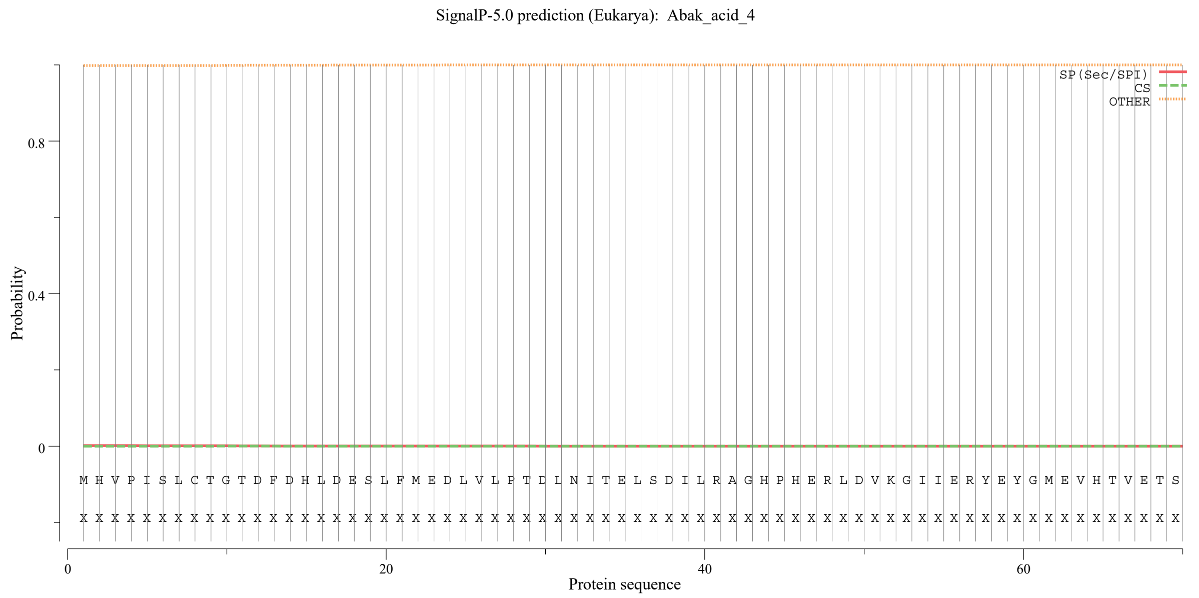

(B)

Figure S3. The predicted signal sequence in lipase with Signal P software taking *Apocrypta bakeri* as an example. (A) An acid lipase with secretion signal, (B) An acid lipase without secretion signal.

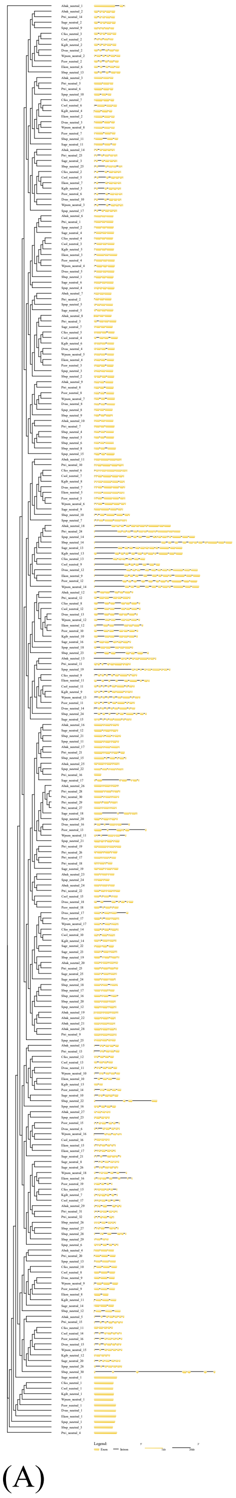

(A)

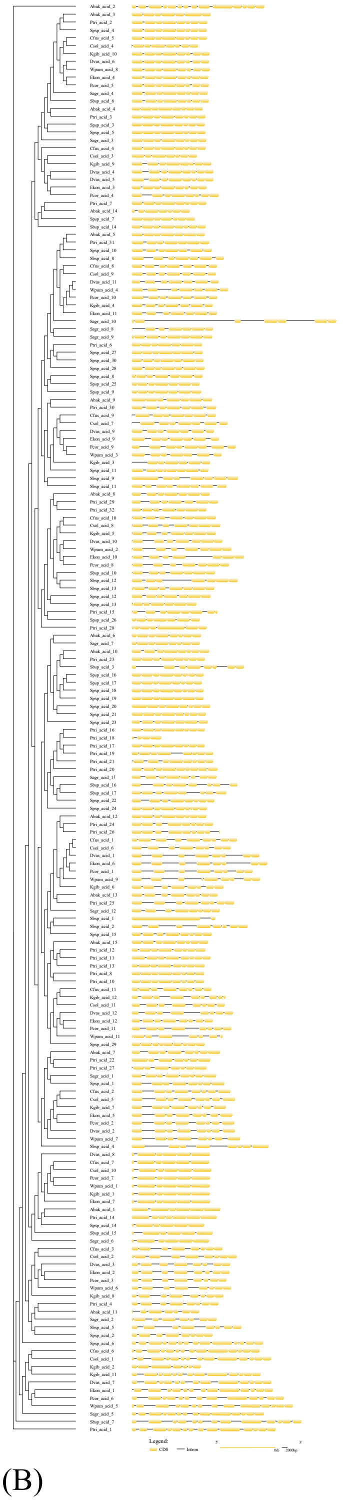

(B)

Figure S4. The exon-intron arrangement in all neutral (A) and acid (B) lipases.

HTBAGGLGLLZPJGHVDFYPNG
NIHLIGHSLGAHVAGFAGYYL
GKVGRITGLDPAGPLFENA
RBAYLKRGDYNVIVVDWSKGA
ACSHGRSYKYFAESINSKCGF
LKNSNFBPSRPTKFII
SLRLDKTDAQFVDVI
PNYVQAAANTRLVGRYLAKLJ
RGTYYLRTNAESPYC
GRXQPGCN
FNSTSCARPPFTCPYSQIQF
YXLYTRPLDTFPJPPEVINT
AVECESWESFLRGKCDGNKEV
HGYLSSGNSEW
RFYLYTRENPDGGZI
SWVQGYMKVTLHADNGVIRNMDLTPSGYMKLEHGT
LAPVICSAGILDPWQWARSDRIEVNIPWLPFENETRCYDELGCLNITRSW
SWAYDMDVLQPRSLCFFWCNDRLYVNSVVVDVMELPGRGKRETDFSSKLC
GKVEAVEITWDYQTSMFNPLTWRLLHKPKAYIDSLTIKSLEFHNEITVCP
AEPCSRRCPSGPRIAPRSGELLVMGQHTPKGSRGSFCLTSYDPPYCPKYH

(A)

HGLLGSSDDWVLLGPNKSLAYJLADAGYDVWLGNARGNTYS
YIGHSMGTTIFYVMLSEKPEYNDKIKAMF
DPKFWBFSWHEMGIYDLPAMIDYILNLTG
LIRKYGYPAEEHTVTTEDGYILTLHRIPG
YNSTEPPEYBLSNITVPVALFYSENDWL
PLILSHFPAGTSVKTFJHYAQ
ADPKDVZKLYSKLPNVIGKYKVPDEKFNHJDFLWAKDAPKLVYDKJLSJM
SGKFRQYDYGLEGNL
SLAPVAFTTHL
QPICSNLLFLIAGFB
PKSPKAPGKPVVFJQ
RSPLIKFIVRFYILMEWGSAYCNIHQWFPRNRLQAKALGTLIRNTPGTLT
YITDDSNIRDLKNTVMNAWGFMMTMVLPTFTIKNFSEMMDHPDVFLDTAQ
LCDNDPCEVNKPKIAVAYILVDRGYDVWLGIGNSSIAENTLSNEENERKR
GIYEFLPRNAF
KEDVLALLNRLPNAEAREVPHERFSHLDF
TVTIIVGIDTYFDTSGRNLYQLNILSEYELERYAFPPVLPTSESEIDEQD
LERVAQYILASTQAEELTVMGHFNGSVQFFGMTAPRIIEEE
RSHVTLSP
SPIFQVLSRISKPLYLLIRLI

(B)

Figure S5. The sequence of 20 motifs used in the phylogenetic tree of neutral lipases (A) and acid lipases (B). The motifs with catalytic triad were circled by red rectangles.
