# Supplementary material for: Genome-Wide Identification and Analysis of Lipases in Fig Wasps (Chalcidoidea, Hymenoptera)
Source: Insects. 2022 Apr 24;13(5):407. doi: 10.3390/insects13050407 (PMC9143690; doi:10.3390/insects13050407)
Supplement: Supplementary file 1 [file insects-13-00407-s001.zip › Supplementary tables-final.pdf]

Table S1 Information of 12 fig wasp samples.

Species	Collection locality	Coordinate	Collection date
Eupristina koningsbergeri	Danzhou, Hainan	109°34′50″N, 19°31′15″E	2016/9/14
Platyscapa corneri	Mount Bawang, Hainan	109°5′24″N, 19°7′39″E	2016/7/11
Kradibia gibbosae	Xishuangbanna, Yunnan	101°15′5″N, 21°56′13″E	2016/12/1
Ceratosolen fusciceps	Xishuangbanna, Yunnan	101°15′5″N, 21°56′13″E	2016/12/19
Dolichoris vasculosae	Mount Jianfeng, Hainan	109°43′16″N, 19°28′53″E	2017/4/3
Wiebesia pumilae	Huangshan, Anhui	118°11′39″N, 30°5′50″E	2017/4/10
Apocrypta bakeri	Xinglong, Hainan	110°12′11″N, 18°44′24″E	2017/5/6
Philotrypesis tridentata	Danzhou, Hainan	109°34′50″N, 19°31′15″E	2017/5/4
Sycobia sp.2	Nada, Hainan	109°33′11″N, 19°31′17″E	2017/5/18
Sycophila sp.2	Nada, Hainan	109°33′11″N, 19°31′17″E	2017/6/10
Sycophaga agraensis	Xishuangbanna, Yunnan	101°15′5″N, 21°56′13″E	2017/7/1

Table S2. The gene list of neutral and acid lipases with incomplete neutral lipase triad.

Category	Fig wasp	Gene name	Predicted active sites residues
Neutral lipase	*Kradibia gibbosae*	Kgib_neutral_15	S42-D84-H-
	Dolichoris vasculosae	Dvas_neutral_18	D237-D264-H349
	Eupristina koningsbergeri	Ekon_neutral_17	A158-D220-H251
	Sycophaga agraensis	Sagr_neutral_24	S175-E203-H278
	Sycophaga agraensis	Sagr_neutral_19	S246-D275-Q344
	Sycobia sp.2	Sbsp_neutral_22	S138-D163-H-
	Sycophila sp.2	Spsp_neutral_4	S202-D232-N297
	Sycophila sp.2	Spsp_neutral_15	S156-D190-A268
	Philotrypesis tridentata	Ptri_neutral_16	S164-D185-H-
	Philotrypesis tridentata	Ptri_neutral_32	S37-D66-H-
Acid lipase	*Wiebesia pumilae*	Wpum_acid_11	S145-D317-H-
	Sycophila sp.2	Spsp_acid_29	G189-D343-H393
	Philotrypesis tridentata	Ptri_acid_19	S-D85-H114
	Philotrypesis tridentata	Ptri_acid_6	G183-D360-H389
	Philotrypesis tridentata	Ptri_acid_18	S-D83H114

Table S3. The CAI values of all the lipases in fig wasps.

Category	Name	CAI
**GDSL**	Ekon_GDSL	0.316
	Cfus_GDSL	0.326
	Pcor_GDSL	0.328
	Dvas_GDSL	0.33
	Csol_GDSL	0.332
	Wpum_GDSL	0.358
	Abak_GDSL_1	0.371
	Spsp_GDSL_1	0.38
	Abak_GDSL_2	0.385
	Sbsp_GDSL	0.393
	Ptri_GDSL	0.47
	Spsp_GDSL_2	0.48
**HSL**	Sagr_HSL_1	0.313
	Cfus_HSL	0.333
	Ekon_HSL_2	0.345
	Spsp_HSL	0.351
	Csol_HSL	0.352
	Ptri_HSL	0.365
	Abak_HSL	0.369
	Dvas_HSL	0.391
	Wpum_HSL	0.395
	Pcor_HSL	0.415
	Sbsp_HSL	0.455
	Ekon_HSL_1	0.457
	Kgib_HSL	0.637
**Lipase3**	Sbsp_lipase3_2	0.41
	Ekon_lipase3_2	0.417
	Abak_lipase3_3	0.451
	Wpum_lipase3	0.455
	Dvas_lipase3	0.457
	Pcor_lipase3	0.469
	Abak_lipase3_1	0.476
	Csol_lipase3	0.491
	Ekon_lipase3_1	0.501
	Cfus_lipase3	0.603
	Sagr_lipase3_1	0.619

	Spsp_lipase3	0.634
	Kgib_lipase3	0.679
	Ptri_lipase3	0.69
	Sbsp_lipase3_1	0.71
**Neutral**	Pcor_neutral_13	0.278
	Pcor_neutral_15	0.286
	Csol_neutral_16	0.289
	Dvas_neutral_16	0.291
	Sagr_neutral_5	0.294
	Sagr_neutral_18	0.294
	Sagr_neutral_19	0.294
	Ekon_neutral_15	0.296
	Ekon_neutral_17	0.298
	Dvas_neutral_6	0.303
	Sagr_neutral_25	0.308
	Csol_neutral_6	0.309
	Dvas_neutral_17	0.309
	Sagr_neutral_24	0.311
	Dvas_neutral_3	0.313
	Ekon_neutral_5	0.313
	Csol_neutral_15	0.316
	Kgib_neutral_6	0.316
	Ptri_neutral_2	0.316
	Sagr_neutral_6	0.316
	Ptri_neutral_22	0.317
	Sbsp_neutral_18	0.317
	Kgib_neutral_12	0.318
	Pcor_neutral_5	0.318
	Sagr_neutral_23	0.32
	Spsp_neutral_20	0.32
	Wpum_neutral_18	0.323
	Abak_neutral_24	0.324
	Pcor_neutral_19	0.324
	Sbsp_neutral_13	0.324
	Spsp_neutral_5	0.325
	Wpum_neutral_16	0.325
	Cfus_neutral_1	0.326
	Csol_neutral_10	0.326
	Kgib_neutral_14	0.326

	Wpum_neutral_11	0.326
	Spsp_neutral_4	0.328
	Sbsp_neutral_16	0.33
	Kgib_neutral_4	0.331
	Sagr_neutral_2	0.331
	Wpum_neutral_17	0.331
	Csol_neutral_1	0.333
	Kgib_neutral_7	0.333
	Abak_neutral_7	0.334
	Kgib_neutral_8	0.334
	Dvas_neutral_18	0.336
	Ptri_neutral_9	0.336
	Sagr_neutral_8	0.336
	Abak_neutral_19	0.337
	Pcor_neutral_7	0.337
	Sbsp_neutral_28	0.339
	Wpum_neutral_8	0.339
	Sagr_neutral_21	0.34
	Ekon_neutral_2	0.344
	Sagr_neutral_17	0.345
	Ptri_neutral_30	0.346
	Sagr_neutral_26	0.347
	Sagr_neutral_12	0.348
	Wpum_neutral_6	0.348
	Csol_neutral_17	0.349
	Abak_neutral_23	0.352
	Ptri_neutral_27	0.352
	Wpum_neutral_1	0.352
	Sbsp_neutral_25	0.353
	Spsp_neutral_21	0.353
	Csol_neutral_2	0.355
	Spsp_neutral_8	0.355
	Sbsp_neutral_20	0.356
	Wpum_neutral_7	0.356
	Dvas_neutral_10	0.357
	Abak_neutral_21	0.358
	Ekon_neutral_16	0.359
	Pcor_neutral_1	0.359
	Pcor_neutral_18	0.359

	Wpum_neutral_5	0.36
	Ekon_neutral_4	0.361
	Abak_neutral_9	0.362
	Ekon_neutral_7	0.362
	Pcor_neutral_2	0.362
	Sbsp_neutral_17	0.362
	Abak_neutral_22	0.363
	Dvas_neutral_4	0.363
	Sbsp_neutral_29	0.364
	Spsp_neutral_25	0.364
	Sbsp_neutral_27	0.366
	Ptri_neutral_16	0.368
	Ptri_neutral_25	0.368
	Sagr_neutral_7	0.368
	Ptri_neutral_7	0.37
	Sbsp_neutral_26	0.37
	Spsp_neutral_12	0.37
	Abak_neutral_10	0.371
	Kgib_neutral_15	0.372
	Cfus_neutral_3	0.376
	Spsp_neutral_3	0.376
	Sagr_neutral_22	0.377
	Csol_neutral_7	0.378
	Kgib_neutral_1	0.379
	Abak_neutral_26	0.381
	Pcor_neutral_3	0.383
	Wpum_neutral_3	0.383
	Abak_neutral_17	0.384
	Spsp_neutral_2	0.384
	Pcor_neutral_8	0.385
	Ptri_neutral_8	0.385
	Csol_neutral_4	0.389
	Cfus_neutral_14	0.391
	Abak_neutral_28	0.396
	Dvas_neutral_1	0.396
	Sbsp_neutral_6	0.396
	Abak_neutral_11	0.397
	Ekon_neutral_6	0.397
	Wpum_neutral_2	0.398

	Abak_neutral_20	0.403
	Dvas_neutral_2	0.403
	Pcor_neutral_4	0.404
	Abak_neutral_2	0.405
	Ekon_neutral_12	0.405
	Spsp_neutral_24	0.405
	Wpum_neutral_15	0.405
	Csol_neutral_14	0.407
	Dvas_neutral_8	0.407
	Spsp_neutral_23	0.407
	Abak_neutral_25	0.408
	Dvas_neutral_13	0.413
	Dvas_neutral_15	0.414
	Ptri_neutral_14	0.415
	Sagr_neutral_3	0.417
	Pcor_neutral_16	0.419
	Cfus_neutral_15	0.42
	Csol_neutral_5	0.42
	Sagr_neutral_16	0.421
	Csol_neutral_13	0.422
	Dvas_neutral_5	0.422
	Pcor_neutral_12	0.424
	Abak_neutral_16	0.425
	Abak_neutral_6	0.426
	Ptri_neutral_21	0.427
	Dvas_neutral_7	0.43
	Pcor_neutral_6	0.43
	Abak_neutral_29	0.431
	Sbsp_neutral_22	0.434
	Ptri_neutral_32	0.436
	Pcor_neutral_17	0.437
	Ptri_neutral_1	0.437
	Ptri_neutral_31	0.437
	Abak_neutral_27	0.438
	Spsp_neutral_10	0.44
	Abak_neutral_8	0.445
	Kgib_neutral_5	0.446
	Cfus_neutral_2	0.45
	Abak_neutral_14	0.451

	Abak_neutral_12	0.453
	Cfus_neutral_11	0.454
	Sagr_neutral_9	0.454
	Sbsp_neutral_14	0.455
	Sagr_neutral_4	0.456
	Sagr_neutral_20	0.456
	Kgib_neutral_3	0.457
	Sbsp_neutral_30	0.458
	Ekon_neutral_11	0.459
	Ekon_neutral_1	0.46
	Ptri_neutral_10	0.463
	Spsp_neutral_15	0.463
	Abak_neutral_15	0.465
	Dvas_neutral_11	0.467
	Spsp_neutral_22	0.467
	Sbsp_neutral_11	0.468
	Wpum_neutral_4	0.468
	Abak_neutral_18	0.469
	Ekon_neutral_3	0.47
	Ptri_neutral_28	0.47
	Wpum_neutral_10	0.471
	Ptri_neutral_6	0.474
	Dvas_neutral_14	0.477
	Sbsp_neutral_5	0.477
	Ptri_neutral_5	0.478
	Spsp_neutral_11	0.479
	Ekon_neutral_9	0.48
	Kgib_neutral_2	0.48
	Abak_neutral_13	0.481
	Abak_neutral_3	0.482
	Kgib_neutral_13	0.488
	Ptri_neutral_19	0.489
	Spsp_neutral_9	0.489
	Abak_neutral_5	0.492
	Ptri_neutral_12	0.492
	Ptri_neutral_3	0.493
	Spsp_neutral_26	0.494
	Spsp_neutral_18	0.495
	Ekon_neutral_10	0.497

	Sbsp_neutral_1	0.499
	Csol_neutral_12	0.501
	Sagr_neutral_11	0.504
	Wpum_neutral_14	0.505
	Cfus_neutral_7	0.508
	Ptri_neutral_17	0.514
	Csol_neutral_3	0.515
	Wpum_neutral_12	0.515
	Spsp_neutral_17	0.518
	Abak_neutral_4	0.52
	Cfus_neutral_12	0.527
	Ptri_neutral_15	0.527
	Sbsp_neutral_8	0.527
	Ptri_neutral_26	0.529
	Cfus_neutral_8	0.53
	Sbsp_neutral_2	0.53
	Ptri_neutral_29	0.532
	Sbsp_neutral_4	0.532
	Cfus_neutral_5	0.551
	Spsp_neutral_6	0.551
	Pcor_neutral_14	0.563
	Wpum_neutral_13	0.565
	Cfus_neutral_9	0.568
	Sbsp_neutral_15	0.569
	Spsp_neutral_7	0.571
	Abak_neutral_1	0.572
	Sbsp_neutral_9	0.572
	Spsp_neutral_19	0.572
	Csol_neutral_9	0.576
	Csol_neutral_11	0.587
	Ptri_neutral_18	0.587
	Dvas_neutral_12	0.592
	Pcor_neutral_10	0.599
	Sbsp_neutral_21	0.599
	Sbsp_neutral_10	0.612
	Spsp_neutral_14	0.615
	Dvas_neutral_9	0.619
	Ptri_neutral_23	0.622
	Pcor_neutral_11	0.625

	Sbsp_neutral_19	0.635
	Wpum_neutral_9	0.643
	Sagr_neutral_10	0.646
	Sbsp_neutral_23	0.648
	Csol_neutral_8	0.652
	Sagr_neutral_15	0.653
	Pcor_neutral_9	0.654
	Ptri_neutral_4	0.66
	Cfus_neutral_6	0.663
	Spsp_neutral_13	0.665
	Spsp_neutral_1	0.669
	Ptri_neutral_11	0.67
	Spsp_neutral_16	0.674
	Kgib_neutral_9	0.678
	Kgib_neutral_10	0.679
	Ekon_neutral_8	0.687
	Sbsp_neutral_24	0.693
	Ptri_neutral_24	0.695
	Cfus_neutral_13	0.704
	Cfus_neutral_10	0.706
	Sagr_neutral_13	0.721
	Ptri_neutral_13	0.732
	Cfus_neutral_4	0.737
	Ptri_neutral_20	0.742
	Sagr_neutral_1	0.743
	Sbsp_neutral_12	0.786
	Kgib_neutral_11	0.788
	Sagr_neutral_14	0.795
	Sbsp_neutral_3	0.823
**Acid**	Ekon_acid_10	0.29
	Cfus_acid_10	0.294
	Dvas_acid_10	0.296
	Spsp_acid_27	0.297
	Ekon_acid_4	0.306
	Csol_acid_8	0.308
	Dvas_acid_9	0.308
	Csol_acid_9	0.309
	Ekon_acid_7	0.309
	Ekon_acid_12	0.309

	Pcor_acid_10	0.311
	Cfus_acid_8	0.313
	Dvas_acid_11	0.313
	Ptri_acid_24	0.314
	Ekon_acid_3	0.316
	Ekon_acid_11	0.316
	Pcor_acid_8	0.316
	Sagr_acid_7	0.317
	Abak_acid_6	0.318
	Ptri_acid_26	0.319
	Sbsp_acid_15	0.319
	Kgib_acid_9	0.321
	Sagr_acid_10	0.321
	Spsp_acid_25	0.323
	Spsp_acid_9	0.325
	Dvas_acid_8	0.326
	Dvas_acid_12	0.326
	Sagr_acid_8	0.326
	Kgib_acid_12	0.328
	Kgib_acid_5	0.329
	Ptri_acid_15	0.33
	Csol_acid_4	0.331
	Csol_acid_11	0.331
	Dvas_acid_5	0.332
	Ekon_acid_1	0.333
	Sagr_acid_11	0.333
	Wpum_acid_11	0.333
	Spsp_acid_30	0.334
	Csol_acid_7	0.335
	Ekon_acid_6	0.335
	Wpum_acid_2	0.337
	Dvas_acid_4	0.338
	Sagr_acid_3	0.338
	Sagr_acid_4	0.339
	Cfus_acid_9	0.34
	Kgib_acid_10	0.34
	Pcor_acid_7	0.34
	Ptri_acid_23	0.341
	Csol_acid_3	0.343

	Spsp_acid_14	0.344
	Abak_acid_12	0.345
	Spsp_acid_5	0.345
	Spsp_acid_13	0.345
	Spsp_acid_18	0.345
	Abak_acid_10	0.346
	Spsp_acid_28	0.346
	Spsp_acid_20	0.347
	Sagr_acid_9	0.348
	Dvas_acid_7	0.349
	Sagr_acid_6	0.35
	Spsp_acid_3	0.351
	Wpum_acid_5	0.351
	Cfus_acid_7	0.352
	Wpum_acid_7	0.352
	Dvas_acid_6	0.353
	Kgib_acid_1	0.353
	Ptri_acid_3	0.353
	Sbsp_acid_17	0.353
	Spsp_acid_8	0.353
	Wpum_acid_1	0.353
	Pcor_acid_6	0.354
	Wpum_acid_8	0.356
	Dvas_acid_1	0.357
	Kgib_acid_3	0.357
	Csol_acid_6	0.359
	Spsp_acid_21	0.359
	Wpum_acid_4	0.359
	Kgib_acid_6	0.36
	Pcor_acid_1	0.36
	Ekon_acid_9	0.362
	Spsp_acid_16	0.362
	Kgib_acid_4	0.364
	Spsp_acid_17	0.365
	Spsp_acid_26	0.366
	Abak_acid_1	0.369
	Sbsp_acid_16	0.369
	Abak_acid_15	0.37
	Csol_acid_10	0.371

	Spsp_acid_12	0.371
	Dvas_acid_2	0.374
	Spsp_acid_15	0.374
	Ekon_acid_5	0.375
	Spsp_acid_7	0.375
	Cfus_acid_11	0.376
	Kgib_acid_11	0.376
	Wpum_acid_3	0.377
	Kgib_acid_2	0.379
	Pcor_acid_11	0.38
	Sbsp_acid_11	0.38
	Wpum_acid_9	0.38
	Pcor_acid_4	0.381
	Dvas_acid_3	0.383
	Ptri_acid_14	0.383
	Abak_acid_4	0.384
	Pcor_acid_2	0.386
	Ptri_acid_20	0.386
	Cfus_acid_4	0.389
	Spsp_acid_23	0.389
	Ekon_acid_2	0.39
	Spsp_acid_29	0.393
	Csol_acid_5	0.395
	Wpum_acid_6	0.397
	Sagr_acid_12	0.398
	Ptri_acid_18	0.4
	Kgib_acid_7	0.402
	Spsp_acid_19	0.402
	Ptri_acid_25	0.403
	Ptri_acid_13	0.405
	Csol_acid_1	0.406
	Sagr_acid_5	0.406
	Spsp_acid_4	0.407
	Ptri_acid_9	0.41
	Abak_acid_8	0.411
	Abak_acid_5	0.413
	Abak_acid_13	0.414
	Ptri_acid_12	0.414
	Spsp_acid_10	0.415

	Ptri_acid_11	0.417
	Pcor_acid_3	0.418
	Abak_acid_3	0.42
	Sbsp_acid_8	0.424
	Csol_acid_2	0.425
	Sagr_acid_1	0.425
	Sagr_acid_2	0.428
	Abak_acid_11	0.429
	Spsp_acid_24	0.429
	Abak_acid_14	0.43
	Ptri_acid_16	0.432
	Spsp_acid_22	0.433
	Kgib_acid_8	0.445
	Sbsp_acid_9	0.447
	Abak_acid_2	0.448
	Ptri_acid_5	0.452
	Sbsp_acid_3	0.455
	Ptri_acid_17	0.456
	Sbsp_acid_6	0.458
	Spsp_acid_11	0.458
	Ptri_acid_19	0.46
	Cfus_acid_1	0.461
	Abak_acid_9	0.462
	Ptri_acid_8	0.462
	Ptri_acid_10	0.462
	Spsp_acid_2	0.462
	Cfus_acid_5	0.463
	Sbsp_acid_14	0.466
	Ptri_acid_2	0.47
	Sbsp_acid_10	0.473
	Ptri_acid_28	0.476
	Sbsp_acid_12	0.486
	Abak_acid_7	0.488
	Spsp_acid_6	0.488
	Ptri_acid_31	0.493
	Sbsp_acid_2	0.498
	Sbsp_acid_1	0.505
	Cfus_acid_6	0.507
	Cfus_acid_2	0.508

	Sbsp_acid_13	0.509
	Spsp_acid_1	0.515
	Ptri_acid_4	0.518
	Ptri_acid_22	0.521
	Cfus_acid_3	0.534
	Ptri_acid_21	0.548
	Ptri_acid_32	0.56
	Ptri_acid_27	0.565
	Ptri_acid_29	0.571
	Ptri_acid_6	0.583
	Ptri_acid_1	0.587
	Ptri_acid_30	0.596
	Sbsp_acid_7	0.596
	Ptri_acid_7	0.622
	Sbsp_acid_4	0.656
	Sbsp_acid_5	0.68
